# Comparative Transcriptomic Analysis of Detoxification Enzyme Gene Families in Parent and Offspring Riptortus pedestris After Sublethal Thiamethoxam Treatment

**DOI:** 10.3390/insects17060648

**Published:** 2026-06-19

**Authors:** Sizhu Zhao, Zijie Wang, Simeng Chen, Ruirui Li, Zhengxiao Du, Xing Huang, Haibin Yuan, Shusen Shi, Yuxin Zhou, Yu Gao

**Affiliations:** 1 College of Plant Protection, Jilin Agricultural University, Changchun 130118, China; 2 Jiamusi Branch of Heilongjiang Academy of Agricultural Sciences, Jiamusi 154007, China; 3 Key Laboratory of Soybean Disease and Pest Control, Ministry of Agriculture and Rural Affairs, Changchun 130118, China; 4 State Key Laboratory of Green Pesticide, Guizhou University, Guiyang 550025, China

**Keywords:** detoxification enzyme genes, neonicotinoid, *Riptortus pedestris*, sublethal concentration, thiamethoxam, intergenerational effects

## Abstract

Thiamethoxam is the main neonicotinoid insecticide used for controlling *Riptortus pedestris* (Fabricius) (Hemiptera: Alydidae). However, sublethal concentration stress may induce intergenerational transcriptional memory, leading to transcriptional patterns that may contribute to the intergenerational accumulation of metabolic tolerance, and evaluating only the toxicity of the current generation would underestimate the long-term risk. Therefore, this study investigated the effect of parental exposure on the expression of detoxification enzyme genes in offspring. Using transcriptome sequencing, we systematically identified three detoxification enzyme gene families (cytochrome P450 monooxygenases (CYPs), carboxylesterases (CCEs), and glutathione S-transferases (GSTs)) in *R. pedestris* and compared their differential expression patterns between the parental and filial generations after thiamethoxam treatment at three sublethal concentrations (LC_10_, LC_30_, and LC_50_). In the parental generation, a Theta family GST was consistently upregulated, while in the filial generation, detoxification genes were predominantly downregulated, and the genes upregulated in the parents were not also upregulated in the offspring. Comparisons of parents and offspring at the same concentration revealed that the medium concentration induced the highest number of intergenerationally upregulated genes, exhibiting a non-linear response pattern. These results indicate that parental exposure to sublethal thiamethoxam leaves an intergenerational transcriptional imprint in the offspring, and the transmission pattern involves transcriptional reprogramming rather than simple replication of the parental response, the mechanism of which remains to be determined. This study provides transcriptomic evidence for understanding the metabolic adaptation and intergenerational resistance evolution of *R. pedestris* to thiamethoxam, offering important reference value for field resistance monitoring and rational insecticide application.

## Simple Summary

The bean bug, *Riptortus pedestris*, is a major soybean pest that is often controlled with the insecticide thiamethoxam. However, whether sublethal doses affect the progeny remains unknown. Therefore, in this study, we investigated how parental exposure to low, non-lethal thiamethoxam concentrations influences detoxification gene expression in the treated parents and their untreated offspring. Using transcriptome analysis, we found that surviving parents transmit an intergenerational effect to the next generation. Notably, offspring do not simply replicate the parental gene expression pattern; instead, their detoxification genes undergo transcriptional reprogramming in a dose-dependent manner. These findings identify a previously uncharacterized route through which resistance might accumulate across generations. Consequently, they may assist researchers and specialists in more accurately monitoring resistance evolution, ultimately supporting more sustainable insecticide use.

## 1. Introduction

*Riptortus pedestris* (Fabricius) (Hemiptera: Alydidae) is widely distributed in East and Southeast Asia and is one of the most serious pests of leguminous crops [1,2]. Adults and nymphs of this pest pierce and suck the leaves, tender stems, and pods of soybean plants [3,4,5], directly causing flower bud drop and shriveled seeds, and can also induce soybean ‘stay-green’ syndrome [6,7], characterized by failure of pod filling, thick dark green leaves at the late growth stage, and in severe cases, unfilled pods and complete crop failure [8,9,10]. In recent years, the occurrence frequency and damage intensity of *R. pedestris* have been continuously increasing in the major soybean-producing regions of northern China, seriously threatening the revitalization and development of China’s soybean industry [11,12]. Currently, chemical control remains the main strategy for managing pest outbreaks, with neonicotinoid insecticides being widely used due to their high efficacy and broad spectrum [13,14].

Thiamethoxam is currently the main neonicotinoid insecticide used against *R. pedestris* [15]. It acts on insect nicotinic acetylcholine receptors to block central nerve transmission, and possesses contact, stomach, and systemic activity [16]. Studies have shown that thiamethoxam significantly suppresses the population growth of the F_1_ generation of *R. pedestris*, reduces female adult longevity and fecundity, and decreases the intrinsic rate of population increase while prolonging the mean generation time [17]. However, long-term use of chemical insecticides often leads to the development of resistance in target pests. Although field populations of *R. pedestris* remain relatively susceptible to thiamethoxam, sublethal concentrations have been shown to significantly upregulate detoxification enzyme activity [18,19,20], indicating a potential risk of metabolic resistance. In many insect pests, metabolic resistance is one of the most important resistance mechanisms [21,22,23], and its development is closely associated with the upregulation of detoxification genes or enhanced enzyme activity [24,25]. The detoxification of xenobiotics in insects mainly involves three gene families: cytochrome P450 monooxygenases (CYPs), carboxylesterases (CCEs), and glutathione S-transferases (GSTs). CYPs are involved in the detoxification and metabolism of plant secondary metabolites, insecticides, environmental pollutants, and other xenobiotic compounds, and enhanced metabolic detoxification capacity is closely linked to their overexpression [26]. GSTs promote the elimination of toxic substances by catalyzing the conjugation of glutathione with electrophilic compounds or enhance insect tolerance by counteracting oxidative stress induced by insecticide exposure [27,28]. CCEs participate in the detoxification of organophosphates, pyrethroids, and carbamates through upregulation, amplification, or mutation [29]. Therefore, systematic identification of detoxification enzyme gene families in *R. pedestris* and analysis of their expression changes under sublethal thiamethoxam stress are of great significance for assessing resistance risk and guiding rational insecticide use. However, evaluating only the toxicity of the current generation would seriously underestimate the long-term contribution of sublethal stress to population resistance evolution; sublethal stress may leave a transcriptional memory in the offspring through epigenetic mechanisms, leading to intergenerational accumulation of resistance even without continuous high selection pressure. Therefore, it is necessary to analyze the effect of parental exposure on the expression of detoxification enzyme genes in offspring from an intergenerational perspective.

Currently, systematic studies on detoxification enzyme families in *R. pedestris* are still lacking. Based on this, the present study used transcriptome sequencing technology to perform genome-wide identification and phylogenetic analysis of CCE, CYP, and GST genes in the parental (F_0_) and filial (F_1_) generations of *R. pedestris* exposed to different sublethal concentrations of thiamethoxam (LC_10_, LC_30_, and LC_50_), and compared their differential expression patterns. The aim was to reveal the molecular response mechanisms to sublethal thiamethoxam stress of *R. pedestris* and its intergenerational effects, thereby providing a theoretical basis for further investigation of *R. pedestris*’ metabolic resistance evolution and field resistance monitoring.

## 2. Materials and Methods

### 2.1. Test Insects

The *R. pedestris* used in this experiment were obtained from the net house of the Soybean Domain Technology Innovation Center at Jilin Agricultural University. They were reared in indoor insect rearing cages (temperature: 24 ± 1 °C; photoperiod: L:D = 16 h:8 h; relative humidity: 80 ± 5%) using soybean plants and soaked soybean seeds (Jiyu 47) as host plants to establish an experimental population as the test insect source [1]. The population was reared for at least three generations (≥F_3_) under the same conditions to ensure genetic stability before use in experiments.

### 2.2. Construction of Sublethal Residual Populations of R. pedestris

Based on the previous bioactivity results of Wang et al. [30], the sublethal concentrations of thiamethoxam for *R. pedestris* adults (3-day-old) were calculated as follows: control (CK, 0 mg/L), LC_10_ = 20 mg/L, LC_30_ = 44 mg/L, and LC_50_ = 77 mg/L. These three concentrations were used as low, medium, and high sublethal treatments, respectively. The parental (F_0_) and filial (F_1_) generations under each treatment were designated as B1/C1 (LC_10_), B2/C2 (LC_30_), and B3/C3 (LC_50_), with A1 representing the untreated control.

Construction of the F_0_ (parental) sublethal residual population: Soybean grains that were soaked until they were a ‘kidney shape’ were immersed in thiamethoxam solutions at concentrations of LC_10_, LC_30_, and LC_50_ for 20 s, then removed and air-dried. Three grains were placed into each self-made rearing tube, and five F_0_ adults (3 days old) that had been starved for 24 h were introduced into each tube. Clear water was used as the control (CK). After 24 h, the surviving *R. pedestris* from each treatment were reared separately in rearing cages under the same conditions as above. These were used for subsequent experiments when the F_0_ experimental populations developed to the appropriate stages.

Construction of the F_1_ (filial) sublethal residual population: Adults that developed from each F_0_ sublethal residual population were paired and reared individually in rearing tubes. Eggs (F_1_) were collected daily from each tube, and 120 eggs were collected per sublethal population. The eggs were placed in Petri dishes lined with moist filter paper and incubated in an artificial climate chamber. Newly hatched F_1_ nymphs were reared individually in tubes. Mortality at each instar was recorded daily. Upon reaching adulthood, males and females were paired and reared individually, and the number of eggs laid was recorded daily until the death of both individuals. The rearing conditions were the same as those for the F_0_ generation. The F_1_ experimental populations were used for subsequent experiments when they developed to the appropriate stages.

The test insects used were F_0_ and F_1_ adults of *R. pedestris*, taken from the LC_10_, LC_30_, and LC_50_ treatment populations, all of which were healthy 3-day-old adults after eclosion. All test insects were from a multi-generation standardized laboratory population fed with fresh soybean pods, and only individuals that were actively feeding, normally moving, and without obvious deformities or signs of disease were selected. Adults from each treatment and the control (CK) were starved for 24 h. For each treatment, three *R. pedestris* adults (3 days old) of similar body weights were selected, and three biological replicates were used. After treatment, the adults were dissected to remove the digestive tract, which was then immediately frozen in liquid nitrogen and stored at −80 °C in a labeled freezer for subsequent total RNA extraction.

### 2.3. Sample Preparation, High-Throughput Sequencing, and Transcriptome Sequencing

Total RNA was extracted using TRIzol^®^ reagent (Invitrogen, Carlsbad, CA, USA), strictly following the manufacturer’s instructions. RNA concentration and purity were determined using a NanoDrop 2000 spectrophotometer (IMPLEN, Westlake Village, CA, USA). RNA integrity was assessed using 1% agarose gel electrophoresis and an Agilent 2100 Bioanalyzer (Santa Clara, CA, USA). Only samples with an RNA integrity number (RIN) > 8.0 were selected for subsequent analysis. Qualified RNA samples were sent to Sangon Biotech Co., Ltd. (Shanghai, China), for library preparation and subjected to paired-end sequencing (PE150) on an Illumina HiSeq 2500 platform (San Diego, CA, USA). For each treatment group (F_0_ and F_1_ generations of CK, LC_10_, LC_30_, and LC_50_ groups), three biological replicates were prepared. Each replicate consisted of three pooled 3-day-old *R. pedestris* adults. A total of 21 libraries (7 groups × 3 replicates) were constructed.

### 2.4. RNA Extraction, Transcriptome Processing, and Differentially Expressed Gene Identification

The quality control software Trimmomatic (v0.36) was used to filter out adapter sequences, reads containing unknown bases (N), and low-quality reads (including removal of low-quality bases from the 3′ to 5′ and 5′ to 3′ directions, i.e., Q value < 20) from the raw reads, obtaining high-quality clean data. Q20, Q30, GC content, and duplication levels were calculated. The software Trinity (v2.4.0) was used to assemble, filter, and assemble the high-quality clean data, ultimately obtaining high-quality unigenes.

Clean reads were aligned to the de novo transcriptome assembly using Bowtie2 (v2.3.2). Gene expression levels were quantified as TPM using Salmon (v0.8.2). Differential expression analysis was performed with DESeq2 (v1.12.4) in R (v3.5.1). Normalization was conducted using the median-of-ratios method. Multiple testing correction was applied using the Benjamini–Hochberg procedure to control the false discovery rate (FDR). Genes with a q-value < 0.05 and |log_2_(fold change)| > 1 were considered significantly differentially expressed.

Based on the transcriptome gene annotation results of adult insects, searches were performed using keywords such as ‘CCEs’, ‘CYP’, ‘GST’, ‘Glutathione transferases’, ‘Carboxyl/cholinesterases’, and ‘Cytochromes P450’ to obtain the corresponding unigene sequences. ORF Finder (https://www.ncbi.nlm.nih.gov/orffinder/, accessed on 1 April 2026) was used to obtain the corresponding amino acid sequences. Sequence comparison was performed using NCBI-BLASTp (https://blast.ncbi.nlm.nih.gov/Blast.cgi/, accessed on 2 April 2026) to further identify CCE, CYP, and GST genes in *R. pedestris*. Meanwhile, to systematically identify key response genes in the parental and filial generations of *R. pedestris* at different concentrations, differential expression analysis was performed based on the raw count data of differentially expressed genes. The criteria for defining differentially expressed genes (DEGs) were a q-value < 0.05 and |log_2_(fold change)| > 1. These thresholds were chosen to balance statistical rigor and biological relevance: the stringent q-value (<0.05) effectively controls the false discovery rate in multiple tests, while the |log_2_FC| > 1 cutoff focuses on genes that may mediate significant transcriptional responses of CCEs, CYPs, and GSTs; this approach is commonly used in insect transcriptome studies. Pairwise comparisons were performed for all three concentration groups as follows: B1 vs. A1, B2 vs. A1, B3 vs. A1, C1 vs. A1, C2 vs. A1, C3 vs. A1, C1 vs. B1, C2 vs. B2, and C3 vs. B3.

### 2.5. Phylogenetic Analysis of CCEs, CYPs, and GSTs in R. pedestris

First, the amino acid sequences of the six closely related species mentioned above were obtained from published literature. Using the BLAST tool (v2.12.0) in TBtools software (v3.5.1), these sequences were used as queries to perform a reverse BLAST search (BLASTp) against the local gene database of *R. pedestris* to preliminarily screen for possible homologous sequences. Subsequently, using the preliminarily screened *R. pedestris* amino acid sequences as queries, a second reverse BLAST search was performed against the amino acid sequences of the target genes in this study to further confirm homology. To further ensure the reliability of the sequences, the *R. pedestris* amino acid sequences obtained from the second reverse screening and the closely related species gene sequences obtained from the first reverse screening were submitted separately to the NCBI database for BLASTp alignment. For each query, the top 20 BLAST hits (e value < 1 × 10^−5^) were retained, provided that the hit descriptions corresponded to known detoxification enzyme families. No strict percent identity cutoff was applied, allowing for the inclusion of potentially divergent paralogs. All candidate sequences were subsequently validated by phylogenetic analysis (see below) and conserved domain searches.

The filtered *R. pedestris* amino acid sequences were combined with the amino acid sequences of the six closely related species to construct a multiple sequence alignment file. A phylogenetic tree was constructed using TBtools software. The filtered amino acid sequences of *R. pedestris* CCEs, CYPs, and GSTs were used for homologous tree construction together with the amino acid sequences of CCEs, CYPs, and GSTs from other insects. The bootstrap number was set to 10,000, and the other parameters were set to ‘Auto’. The resulting phylogenetic trees were visualized and beautified using FigTree v1.4.4 software.

## 3. Results

### 3.1. Transcriptome Sequencing and Assembly Quality Assessment

Transcriptome sequencing was performed on 21 libraries (seven treatment groups × three biological replicates) using the Illumina HiSeq 2500 platform. An average of 52.90 million raw reads per sample were obtained. The sequencing quality was high, with Q30 values > 94% and GC contents ranging from 39% to 48% (Table S1).

All clean reads were assembled de novo using Trinity, yielding 679,731 transcripts and 385,902 unigenes. The unigene N50 was 504 bp, with 26,790 unigenes (6.94%) being ≥1000 bp (Table S2). Functional annotation against the NR, NT, Swiss-Prot, KOG, KEGG, GO, PFAM, and CDD databases resulted in 94,143 unigenes annotated in NR (24.4% of total unigenes), with longer sequences showing higher annotation rates (Table S2).

These quality metrics (Q30 > 94%, unigene N50 = 504 bp) meet the standards for reliable downstream differential expression analysis (Table S3).

### 3.2. Identification of CCE, CYP, and GST Gene Families in R. pedestris

**Figure 1:**
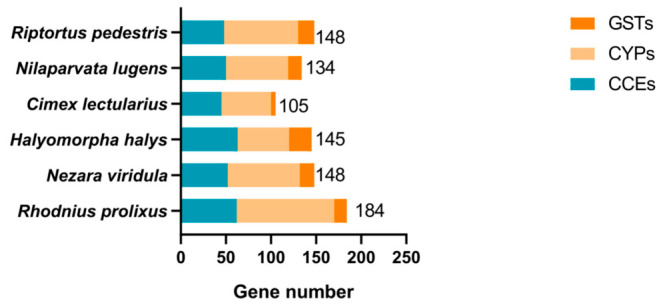
Comparison of the number distribution of detoxification enzyme-related gene families across different insect species.

In this study, we selected five previously reported hemipteran species [31] (*Nezara viridula* L., *Halyomorpha halys* (Stål), *Rhodnius prolixus* (Stål), *Cimex lectularius* L., and *Nilaparvata lugens* (Stål)) as closely related reference species and *Bemisia tabaci* Gennadius as an outgroup to perform phylogenetic analysis of detoxification-related gene families. According to the total number of members of each gene superfamily, 148 detoxification-related genes were identified in *N. viridula*, 145 in *H. halys*, 184 in *R. prolixus*, 105 in *C. lectularius*, and 134 in *N. lugens*. The CYP superfamily had the highest number of genes in all species (except *H. halys*), followed by CCEs, while GSTs represented the smallest family (Figure 1).

From the transcriptome data of *R. pedestris*, a total of 48 CCEs, 82 CYPs, and 18 GSTs were identified and screened. Among the 48 CCEs, named according to their phylogenetic relationship with *N. viridula* (Nvir) in the phylogenetic tree, the encoded amino acid lengths ranged from 61 to 981 aa, with 21 CCEs being full-length. The amino acid-level homology of the identified CCE genes with those of known insects ranged from 25.00% to 94.81%. Among the 82 CYPs, also named based on their phylogenetic relationship with *N. viridula*, the encoded amino acid lengths ranged from 76 to 524 aa, with 42 CYPs being full-length. The amino acid-level homology of the identified CYP genes with those of known insects ranged from 26.61% to 100.00%. Among the 18 GSTs, named according to their phylogenetic relationship with *N. viridula*, the encoded amino acid lengths ranged from 29 to 217 aa, with three GSTs being full-length. The amino acid-level homology of the identified GST genes with those of known insects ranged from 44.63% to 100.00% (summarized in Table 1; detailed in Supplementary Table S4).

### 3.3. Homology Analysis of CCEs, CYPs, and GSTs in R. pedestris

**Figure 2:**
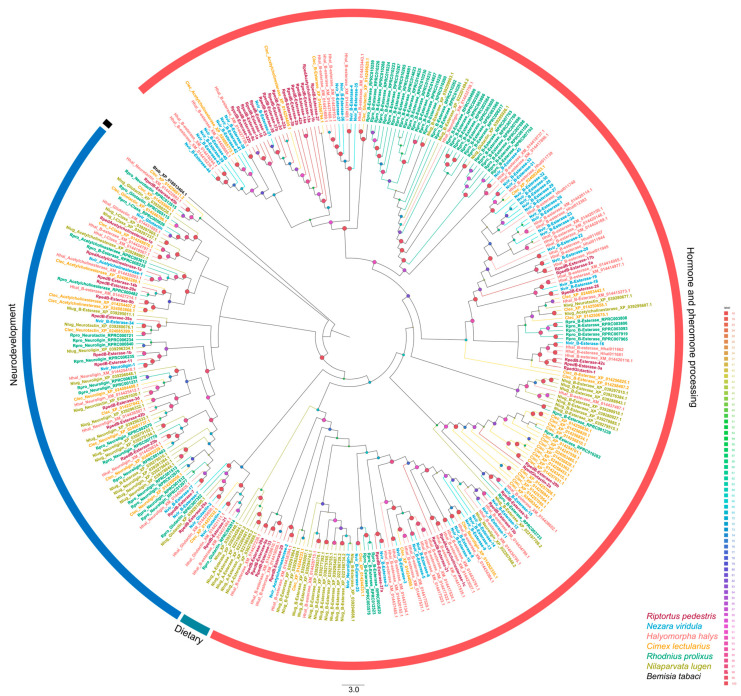
Phylogenetic analysis of CCEs from *R. pedestris* and other representative insect species. The sequences used to construct the phylogenetic tree were derived from the following seven species: *R. pedestris* (Rped), *Nezara viridula* (Nvir), *Halyomorpha halys* (Hhal), *Cimex lectularius* (Clec), *Rhodnius prolixus* (Rpro), *Nilaparvata lugens* (Nlug), and *Bemisia tabaci* (Btab). Colored circles at the nodes indicate bootstrap support values (10,000 replicates).

Based on the phylogenetic tree constructed using the amino acid sequences of CCEs from *R. pedestris* and other insects, the genes in the tree were classified into three major functional categories: Dietary, Hormone and Pheromone Processing, and Neurodevelopment. Among these, 15 *R. pedestris* CCE genes fell into the ‘Neurodevelopment branch’, none were found in the ‘Dietary branch’, and 33 were clustered in the ‘Hormone and Pheromone Processing branch’. *R. pedestris* shared extremely high homology with pentatomid insects such as *Nezara viridula* and *Halyomorpha halys* at this gene locus, with very high clustering support. This gene family exhibits lineage-specific conservation within Hemiptera and is concentrated in the ‘Hormone and Pheromone Processing’ pathway branch (Figure 2).

**Figure 3:**
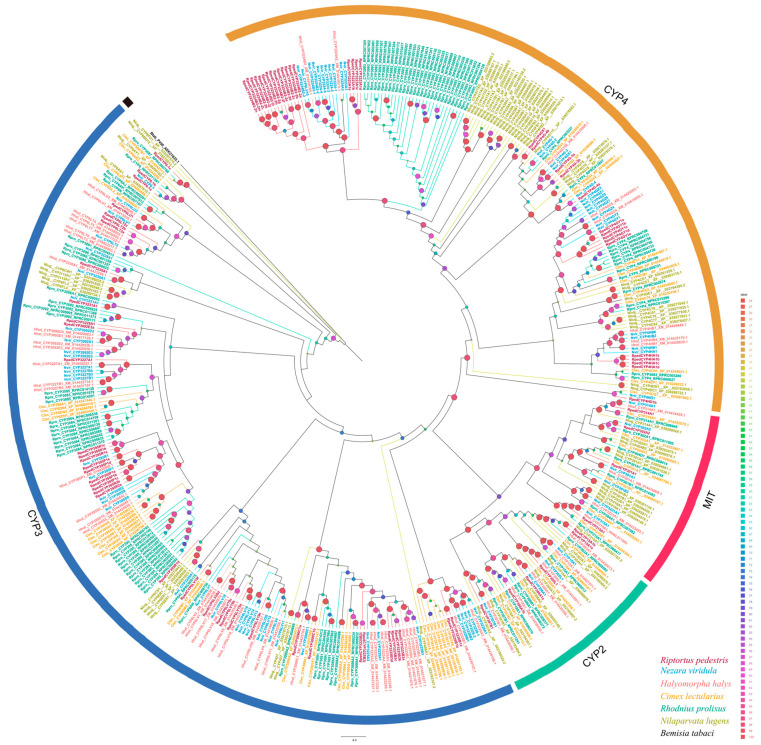
Phylogenetic analysis of CYPs from *R. pedestris* and other representative insect species. The sequences used to construct the phylogenetic tree were derived from the following seven species: *R. pedestris* (Rped), *Nezara viridula* (Nvir), *Halyomorpha halys* (Hhal), *Cimex lectularius* (Clec), *Rhodnius prolixus* (Rpro), *Nilaparvata lugens* (Nlug), and *Bemisia tabaci* (Btab). Colored circles at the nodes indicate bootstrap support values (10,000 replicates).

Based on the phylogenetic tree constructed using the amino acid sequences of CYPs from *R. pedestris* and other insects, the CYP450 genes of *R. pedestris* fully cover the four core CYP450 families of insects (CYP2, CYP3, CYP4, and MIT (mitochondrial CYPs)). In the CYP3, CYP2, MIT, and CYP4 branches, there were 39, six, four, and 33 *R. pedestris* CYP genes, respectively. Among these, the CYP3 family exhibited significant species-specific expansion and is the core gene family for detoxification metabolism and insecticide resistance in *R. pedestris*. The phylogenetic relationships indicate that the CYP450 genes of *R. pedestris* share the highest homology with those of closely related pentatomids such as *Nezara viridula* and *Halyomorpha halys*, preferentially clustering into the same evolutionary clade, demonstrating high conservation within the Pentatomomorpha insects. They clustered into a larger clade with heteropteran groups such as *Cimex lectularius* and *Rhodnius prolixus* but are clearly differentiated from more distantly related hemipteran groups such as *Nilaparvata lugens* and *Bemisia tabaci* (Figure 3).

**Figure 4:**
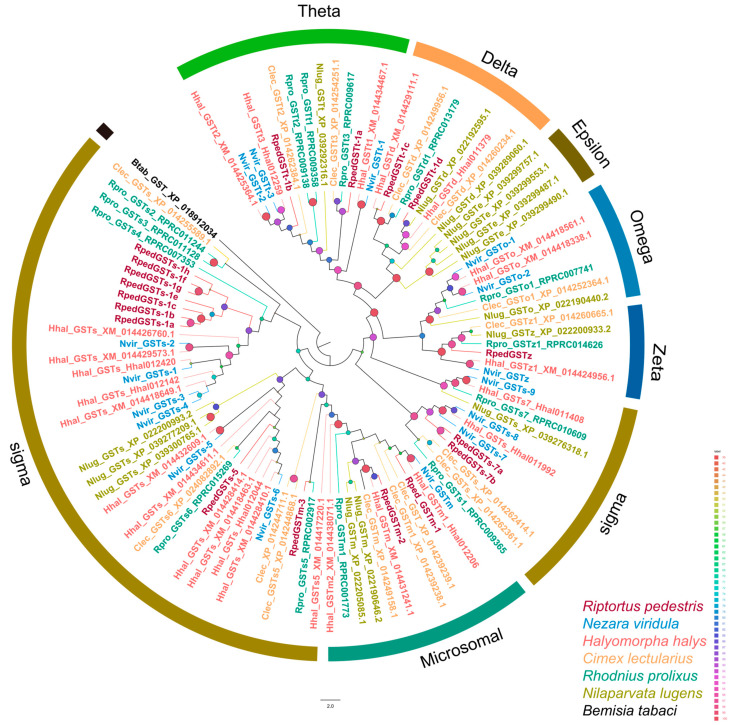
Phylogenetic analysis of GSTs from *R. pedestris* and other representative insect species. The sequences used to construct the phylogenetic tree were derived from the following seven species: *R. pedestris* (Rped), *Nezara viridula* (Nvir), *Halyomorpha halys* (Hhal), *Cimex lectularius* (Clec), *Rhodnius prolixus* (Rpro), *Nilaparvata lugens* (Nlug), and *Bemisia tabaci* (Btab). Colored circles at the nodes indicate bootstrap support values (10,000 replicates).

Based on the phylogenetic tree constructed using the amino acid sequences of GSTs from *R. pedestris* and other insects, the GST genes of *R. pedestris* fully cover the seven classic GST families of insects (Delta, Epsilon, Omega, Zeta, Sigma, Theta, and Microsomal). In the Sigma branch, 11 *R. pedestris* GST genes were found; in the Delta, Theta, and Microsomal branches, there were two GST genes each; no GSTs were found in the Epsilon or Omega branches; and in the Zeta branch, there was one GST gene. The phylogenetic relationships indicate that the GST genes of *R. pedestris* share the highest homology with those of closely related pentatomids such as *Nezara viridula* and *Halyomorpha halys*, preferentially clustering into the same evolutionary clade, demonstrating high conservation within the Pentatomomorpha insects. They clustered into a larger clade with heteropteran groups such as *Cimex lectularius* and *Rhodnius prolixus* but are clearly differentiated from more distantly related hemipteran groups such as *Nilaparvata lugens* and *Bemisia tabaci* (Figure 4).

### 3.4. Differential Gene Expression Between the Parental and Filial Generations of R. pedestris

In the transcriptome data of the parental generation of *R. pedestris*, a total of 70 differentially expressed genes (DEGs) were identified, including 24 CCE genes, 37 CYP genes, and nine GST genes. A1 is the blank control, B1 represents parental adults treated with LC_10_, B2 represents parental adults treated with LC_30_, and B3 represents parental adults treated with LC_50_. The main upregulated and downregulated candidate detoxification genes in both generations are summarized in Table 2.

**Figure 5:**
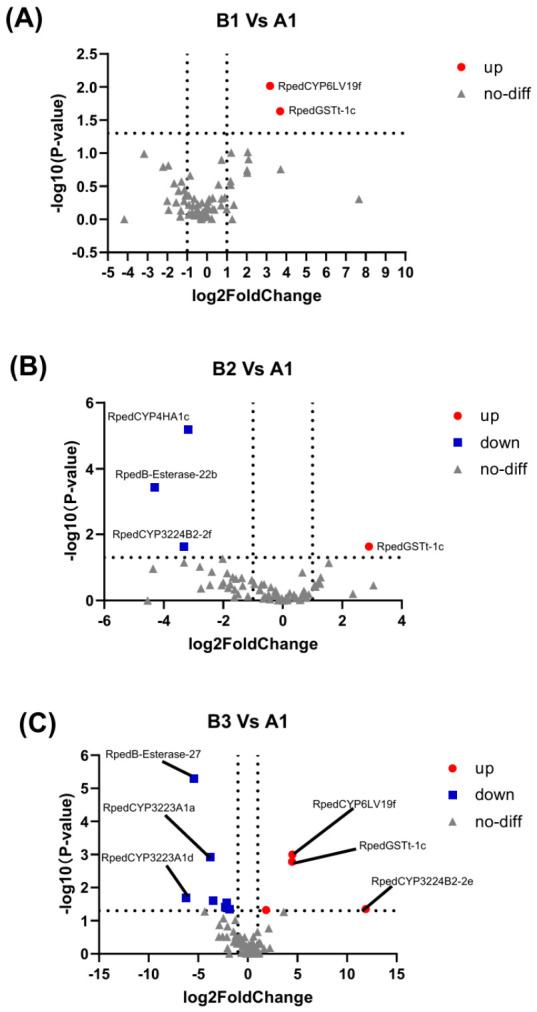
Volcano plots showing differentially expressed genes in the parental generation compared with the control (B vs. A1). (**A**) B1 (LC_10_) vs. A1 (CK); (**B**) B2 (LC_30_) vs. A1 (CK); (**C**) B3 (LC_50_) vs. A1 (CK). The x-axis represents log_2_ (fold change) and the y-axis represents −log_10_(q-value). Red dots indicate significantly upregulated genes (|log_2_FC| > 1, q < 0.05), blue dots indicate significantly downregulated genes (|log_2_FC| < −1, q < 0.05), and gray dots represent non-significant differentially expressed genes. The horizontal dashed line indicates the significance threshold (−log~10~(0.05) ≈ 1.3), and the vertical dashed lines indicate the fold-change thresholds (|log~2~FC| = 1).

In the B1 vs. A1 comparison, two genes were upregulated: *RpedGSTt-1c* and *RpedCYP6LV19f*. In the B2 vs. A1 comparison, one gene was upregulated (*RpedGSTt-1c*) and three genes were downregulated (*RpedCYP4HA1c*, *RpedCYP3224B2-2f*, and *RpedB-Esterase-22b*). In the B3 vs. A1 comparison, four genes were upregulated (*RpedCYP3224B2-2e*, *RpedGSTt-1c*, *RpedCYP6LV19f*, and *RpedB-Esterase-3b*) and seven genes were downregulated (*RpedCYP3223A1d*, *RpedCYP4HA1c*, *RpedB-Esterase-29*, *RpedCYP4GY1e*, *RpedCYP4G-3a*, *RpedB-Esterase-27*, and *RpedCYP3223A1a*). The common upregulated genes across comparisons were *RpedGSTt-1c* and *RpedCYP6LV19f*, while the common downregulated gene was *RpedCYP4HA1c* (Figure 5).

**Figure 6:**
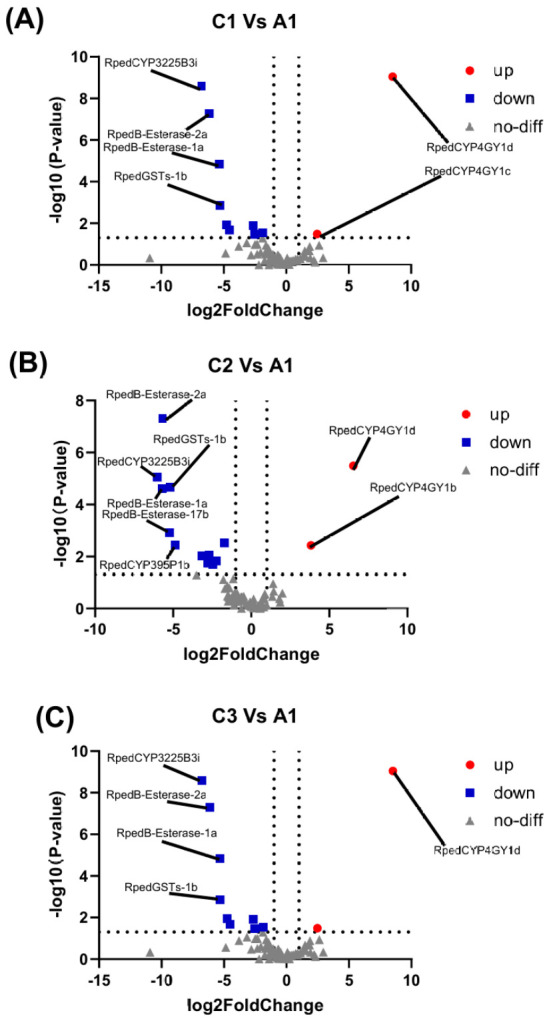
Volcano plots showing differentially expressed genes in the filial generation compared with the control (C vs. A1). (**A**) C1 (LC_10_ offspring) vs. A1 (CK); (**B**) C2 (LC_30_ offspring) vs. A1 (CK); (**C**) C3 (LC_50_ offspring) vs. A1 (CK). The x-axis represents log_2_ (fold change) and the y-axis represents −log_10_(q-value). Red dots indicate significantly upregulated genes (|log_2_FC| > 1, q < 0.05), blue dots indicate significantly downregulated genes (|log_2_FC| < −1, q < 0.05), and gray dots represent non-significant differentially expressed genes. The horizontal dashed line indicates the significance threshold (−log~10~(0.05) ≈ 1.3), and the vertical dashed lines indicate the fold-change thresholds (|log~2~FC| = 1).

In the transcriptome data of the filial generation of *R. pedestris*, a total of 78 differentially expressed genes were identified, including 24 CCE genes, 45 CYP genes, and nine GST genes. C1 represents filial adults derived from the LC_10_ treatment, C2 represents filial adults derived from the LC_30_ treatment, and C3 represents filial adults derived from the LC_50_ treatment. In the C1 vs. A1 comparison, two genes were upregulated (*RpedCYP4GY1d* and *RpedCYP4GY1c*) and nine genes were downregulated (*RpedGSTt-1a*, *RpedCYP314A1*, *RpedGSTs-1b*, *RpedCYP3226B1a*, *RpedCYP3225B3i*, *RpedB-Esterase-2a*, *RpedCYP302A2*, *RpedB-Esterase-1a*, and *RpedCYP3090C1b*). In the C2 vs. A1 comparison, two genes were upregulated (*RpedCYP4GY1d* and *RpedCYP4GY1b*) and twelve genes were downregulated (*RpedCYP395R1a*, *RpedB-Esterase-37b*, *RpedGSTs-1c*, *RpedGSTs-1b*, *RpedB-Esterase-17b*, *RpedB-Esterase-2b*, *RpedCYP3226B1a*, *RpedCYP3225B3i*, *RpedB-Esterase-2a*, *RpedB-Esterase-1a*, *RpedCYP3090C1b*, and *RpedCYP395P1b*). In the C3 vs. A1 comparison, one gene was upregulated (*RpedCYP4GY1b*) and twelve genes were downregulated (*RpedCYP395R1a*, *RpedCYP6LV19c*, *RpedCYP3224B2-2k*, *RpedGSTs-1c*, *RpedGSTs-1b*, *RpedGSTt-1d*, *RpedCYP3226B1a*, *RpedCYP3225B3i*, *RpedCYP6-1a*, *RpedB-Esterase-2a*, *RpedB-Esterase-1a*, and *RpedCYP3090C1b*). The common upregulated genes across these comparisons were *RpedCYP4GY1d* and *RpedCYP4GY1b*, while the common downregulated genes (eight in total) were *RpedGSTs-1b*, *RpedCYP3226B1a*, *RpedCYP3225B3i*, *RpedB-Esterase-2a*, *RpedB-Esterase-1a*, *RpedCYP3090C1b*, *RpedGSTs-1c*, and *RpedCYP395R1a* (Figure 6).

**Figure 7:**
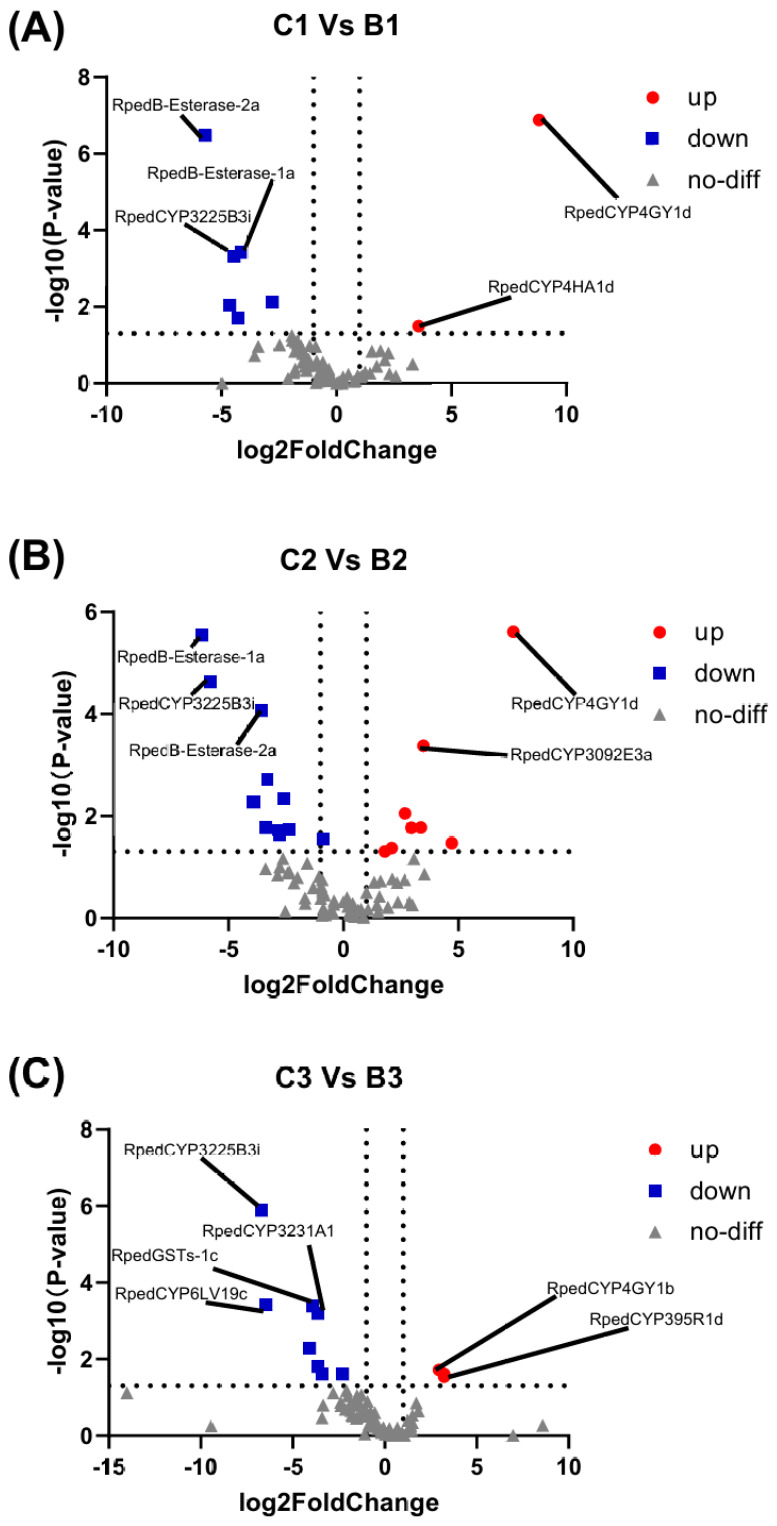
Volcano plots showing differentially expressed genes in direct offspring–parent comparisons (C vs. B). (**A**) C1 vs. B1 (low concentration intergenerational comparison); (**B**) C2 vs. B2 (medium concentration intergenerational comparison); (**C**) C3 vs. B3 (high concentration intergenerational comparison). The x-axis represents log_2_(fold change) and the y-axis represents −log_10_(q-value). Red dots indicate significantly upregulated genes (|log_2_FC| > 1, q < 0.05), blue dots indicate significantly downregulated genes (|log_2_FC| < −1, q < 0.05), and gray dots represent non-significant differentially expressed genes. The horizontal dashed line indicates the significance threshold (−log~10~(0.05) ≈ 1.3), and the vertical dashed lines indicate the fold-change thresholds (|log~2~FC| = 1).

In the transcriptome data comparing the filial generation to the parental generation of *R. pedestris*, a total of 78 differentially expressed genes were identified. In the C1 vs. B1 comparison, two genes were upregulated (*RpedCYP4GY1d* and *RpedCYP4HA1d*) and six genes were downregulated (*RpedGSTs-1b*, *RpedCYP3225B3i*, *RpedB-Esterase-2a*, *RpedCYP302A2*, *RpedB-Esterase-1a*, and *RpedCYP3090C1b*). In the C2 vs. B2 comparison, eight genes were upregulated (*RpedCYP4GY1d*, *RpedB-Esterase-9a*, *RpedCYP395R1b*, *RpedCYP3092E3a*, *RpedCYP4GY1b*, *RpedB-Esterase-38*, *RpedCYP3223A1b*, and *RpedCYP395P1d*) and eleven genes were downregulated (*RpedCYP395R1a*, *RpedB-Esterase-37b*, *RpedCYP3231A1*, *RpedGSTs-1c*, *RpedGSTs-1b*, *RpedB-Esterase-2b*, *RpedCYP3226B1a*, *RpedCYP3225B3i*, *RpedB-Esterase-2a*, *RpedB-Esterase-14b*, and *RpedB-Esterase-1a*). In the C3 vs. B3 comparison, three genes were upregulated (*RpedCYP395R1d*, *RpedCYP4GY1b*, and *RpedCYP395P1d*) and eight genes were downregulated (*RpedCYP6LV19c*, *RpedCYP3224B2-2a*, *RpedCYP3231A1*, *RpedGSTs-1c*, *RpedGSTs-1b*, *RpedCYP6LV19b*, *RpedCYP3225B3i*, and *RpedB-Esterase-2a*) (Figure 7).

### 3.5. Expression Pattern Analysis of CCEs, CYPs, and GSTs in R. pedestris

**Figure 8:**
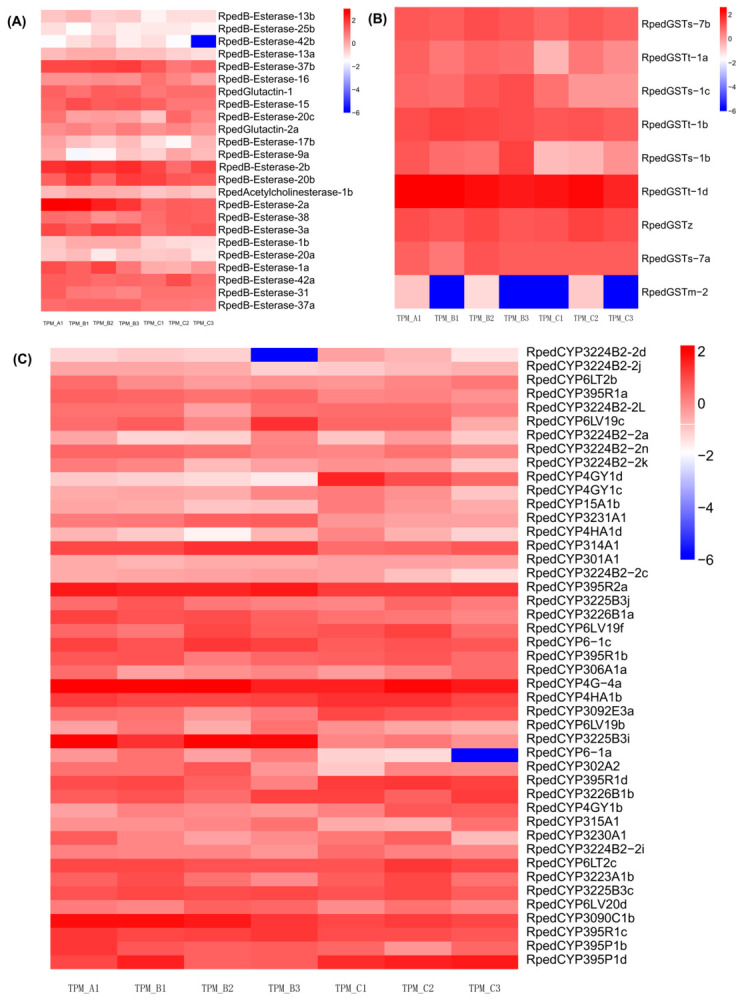
Heatmaps showing the expression profiles of differentially expressed detoxification enzyme genes in *R. pedestris* after treatment with three concentrations of thiamethoxam (LC_10_, LC_30_, and LC_50_) in the parental (F_0_) and filial (F_1_) generations: (**A**) carboxylesterase (CCE) genes; (**B**) glutathione S-transferase (GST) genes; (**C**) cytochrome P450 (CYP) genes. The color scale from blue (low expression) to red (high expression) represents normalized expression values (log_10_ counts), visualizing changes in gene expression levels under different experimental conditions. The x-axis indicates the control (A1) and the different concentration treatments in the parental (F_0_) and filial (F_1_) generations; the y-axis lists the differentially expressed genes for each gene family.

The clustering heatmap of 24 CCE genes in the parental and filial generations of *R. pedestris* shows that *RpedB-Esterase-37b*, *RpedB-Esterase-2b*, and *RpedB-Esterase-2a* exhibited relatively high expression levels in both generations. The clustering heatmap of 45 CYP genes revealed that *RpedCYP395R2a*, *RpedCYP4G-4a, RpedCYP3225B3i*, *RpedCYP3090C1b*, and *RpedCYP395P1d* all displayed high expression levels in both generations. The clustering heatmap of nine GST genes showed that *RpedGSTt-1d* had a high expression level, while *RpedGSTm-2* had a low expression level in the parental and filial generations (Figure 8).

### 3.6. GO and KEGG Enrichment Analyses

To characterize the biological functions and pathways of the differentially expressed genes (DEGs), we performed Gene Ontology (GO) and Kyoto Encyclopedia of Genes and Genomes (KEGG) enrichment analyses. The top enriched terms for each comparison are summarized in Table 3, and the full results are provided in Supplementary Figures S1–S18.

In the parental generation, B1 vs. A1 (LC_10_) was enriched in ‘cytosolic ribosome’ (GO) and ‘synthesis and degradation of ketone bodies’ (KEGG). B2 vs. A1 (LC_30_) was enriched in ‘inner mitochondrial membrane protein complex’ (GO) and ‘oxidative phosphorylation’ (KEGG). B3 vs. A1 (LC_50_) was uniquely enriched in ‘negative regulation of potassium ion transport’ (GO) and the ‘Toll and Imd signaling pathway’ (KEGG).

In the offspring comparisons (C1, C2, and C3 vs. A1), all three groups showed enrichment in ‘cardiac muscle contraction’ (KEGG) and the following distinct GO terms: ‘myofibril’ for C1 vs. A1, ‘ribosome assembly’ for C2 vs. A1, and ‘sperm fibrous sheath’ for C3 vs. A1.

In the intergenerational comparisons, C1 vs. B1 was enriched in ‘response to organic cyclic compound’ (GO) and ‘ECM–receptor interaction’ (KEGG). C2 vs. B2 was uniquely enriched in ‘kininogen binding’ (GO) and ‘cardiac muscle contraction’ (KEGG), consistent with the highest number of intergenerationally upregulated genes in this group. C3 vs. B3 was enriched in ‘protein-phosphocysteine-sugar phosphotransferase activity’ (GO) and ‘carbon metabolism’ (KEGG).

## 4. Discussion

Based on transcriptome data, this study identified 48 CCE genes, 82 CYP genes, and 18 GST genes in *R. pedestris*. The quantitative distribution pattern (CYP > CCE > GST) is consistent with that of several hemipteran species [21] but differs from that of *Drosophila melanogaster* (GST > CCE) [24], suggesting that the detoxification enzyme gene families in Hemiptera exhibit lineage-specific expansion patterns. Furthermore, phylogenetic analysis revealed that the CCEs of *R. pedestris* are mainly enriched in the ‘Hormone and Pheromone Processing’ pathway (33 genes) and are absent from the ‘Dietary’ branch; the CYPs fully cover the four core families (CYP2, CYP3, CYP4, and MIT), with the CYP3 family showing significant expansion (39 genes); similarly, the GSTs cover seven classic families, with the Sigma family exhibiting the most pronounced expansion (11 genes). Notably, all three gene families share high homology with those of pentatomid insects such as *Nezara viridula* and *Halyomorpha halys*, displaying an evolutionary feature of ‘conserved core functions and expansion of detoxification- and stress-related families’. Thus, the composition of these gene families laid a molecular foundation for the subsequent transcriptional expression analysis.

A major highlight of this study is the simultaneous establishment of parental (F_0_) and filial (F_1_) experimental populations under sublethal thiamethoxam concentrations, and the use of comparative transcriptomics to reveal the intergenerational differential expression patterns of detoxification enzyme genes. First, at the transcriptional level in the parental generation (F_0_), a total of 70 differentially expressed genes were identified. Among them, *RpedGSTt-1c* (Theta family GST) was consistently upregulated at all three concentrations (LC_10_, LC_30_, and LC_50_), suggesting that this gene is an early sensitive marker of the thiamethoxam stress response in *R. pedestris*. This finding is supported by previous studies: GSTs have been confirmed to participate in insecticide detoxification in various insects [27]: for example, *CmGSTd2*, *CmGSTe6*, and *CmGSTe7* in the rice leaffolder, *Cnaphalocrocis medinalis* (Guenée), are rapidly induced after chlorpyrifos exposure [32]; the constitutive high expression of *GSTd2* and *GSTe2* in pyrethroid-resistant populations of *Anopheles sinensis* Wiedemann is necessary to maintain high resistance levels [33]; and *PxGSTs1* in the diamondback moth mediates resistance to multiple insecticides through direct detoxification and antioxidant mechanisms and is finely regulated by miRNA [34]. Collectively, these studies indicate that transcriptional upregulation of GSTs is a common strategy for insects to cope with insecticide stress. Moreover, *RpedCYP6LV19f* (CYP3 family) was upregulated at low and high concentrations in the parental generation, whereas *RpedCYP4HA1c* (CYP4 family) was downregulated in all treatments, which may suggest functional differentiation between different CYP family members regarding metabolic functions: the CYP3 family is often associated with xenobiotic detoxification, while the CYP4 family is thought to be more involved in endogenous metabolism [31]. Furthermore, KEGG enrichment analysis showed that B3 vs. A1 (LC_50_) was uniquely enriched in the Toll and Imd signaling pathway, which may be involved in immune or stress responses to high thiamethoxam exposure. In contrast, the DEGs of B1/B2 were enriched in ribosome and fatty acid metabolism pathways [35,36].

Turning to the filial generation, at the transcriptional level in the filial generation (F_1_), a total of 78 differentially expressed genes were identified, slightly more than in the parental generation, but the expression pattern was markedly different: detoxification genes were predominantly downregulated, with very few upregulated genes, and the only co-upregulated genes were *RpedCYP4GY1d/b* (CYP4 family), while the genes upregulated in the parental generation (*RpedGSTt-1c* and *RpedCYP6LV19f*) were not upregulated in the offspring. Consequently, this result clearly demonstrates that parental exposure to sublethal thiamethoxam concentrations leaves an intergenerational transcriptional imprint in their offspring, and the molecular form of intergenerational transmission is not a simple replication of the parental response pattern but rather the activation of a different detoxification gene network. A similar phenomenon has been reported in mosquito populations selected with permethrin: in the offspring, *CYP6AA7* and *CYP4C52v1* were upregulated, while *CYP6BY3* was downregulated, and these changes spanned larval and adult stages [37]. Thus, the predominantly downregulated transcriptional response in the offspring in this study may reflect a trade-off strategy between energy metabolism and detoxification defense in insects, or transcriptional suppression may involve epigenetic regulation such as DNA methylation and histone modification [38]. We present these interpretations as hypotheses requiring functional validation (e.g., via RNAi or heterologous expression). Interestingly, all offspring groups showed enrichment in oxidative phosphorylation pathways, but detoxification pathways were not significantly enriched, indicating a shift from detoxification in parents to energy metabolism in offspring [36,39]. However, the absence of a statistically significant enrichment for detoxification pathways in the F_1_ generation does not necessarily imply biological irrelevance, as subtle but functionally important changes in individual genes may fall below the detection threshold of pathway-level analysis.

Direct comparisons between the offspring and parental generations (C_1_ vs. B_1_, C_2_ vs. B_2_, and C_3_ vs. B_3_) further revealed the core features of intergenerational expression differences. Specifically, the comparison C_2_ vs. B_2_ showed the highest number of upregulated genes (eight), including *RpedCYP4GY1d*, *RpedCYP4GY1b*, and *RpedCYP395P1d*, whereas C_1_ vs. B_1_ and C_3_ vs. B_3_ had fewer upregulated genes (two and three, respectively). This non-linear response pattern suggests that the medium concentration (LC_30_) may be more effective at inducing the activation of intergenerational detoxification genes, while excessively high or low concentrations may weaken the intergenerational effect due to either excessive toxicity or insufficient stimulation. Moreover, in all offspring-vs-parent comparisons, *RpedGSTs-1b*, *RpedCYP3225B3i*, *RpedB-Esterase-2a*, *RpedB-Esterase-1a*, and *RpedCYP3090C1b* were consistently downregulated. Hence, the sustained low expression of these genes in the offspring may reflect an adaptive adjustment of detoxification capacity or a reconfiguration of metabolic pathways following parental stress. Regarding functional enrichment, all three offspring groups (C1, C2, and C3 vs. A1) were enriched in the ‘cardiac muscle contraction’ (KEGG) pathway, with variable GO terms (myofibril, ribosome assembly, and sperm fibrous sheath). No detoxification pathways were significantly enriched in any filial comparison. It is also worth noting that the lack of statistical significance for detoxification pathways does not rule out biological importance, as individual gene changes may be below the pathway detection threshold. These non-linear, concentration-dependent patterns are typical of insecticide hormesis [40].

Expression pattern heatmap analysis further supported the stability of the observed transcriptional expression patterns. *RpedB-Esterase-37b*, *RpedCYP395R2a*, and *RpedGSTt-1d* maintained relatively high expression levels in the parental and filial generations, suggesting that they may play a sustained and stable constitutive defense role in detoxification metabolism. In contrast, *RpedGSTm-2* showed low expression, indicating functional division between different GST family members: the Theta family may be more involved in basal detoxification, while the Microsomal family may participate in more specific physiological processes. It is important to note that our study did not directly assess thiamethoxam susceptibility (e.g., LC_50_ or survival rates) in the F_1_ generation. Therefore, while the observed transcriptional changes are consistent with enhanced detoxification capacity, we cannot conclude that resistance has evolved. Instead, our results provide a foundation for future hypothesis-driven functional studies (e.g., RNAi, heterologous expression, or transgenic approaches) to test whether the identified genes confer measurable resistance phenotypes.

In summary, this study systematically identified three detoxification enzyme gene families in *R. pedestris* through transcriptome sequencing, and for the first time compared the differential expression patterns between the parental and filial generations after exposure to sublethal thiamethoxam concentrations, revealing the molecular response characteristics and intergenerational effects of *R. pedestris* under thiamethoxam stress. It should be noted that this study adopted a de novo transcriptome assembly strategy, which, although effective in discovering novel transcripts, cannot obtain full-length gene sequences or complete alternative splicing information, and the annotation results relied on homology comparison with closely related species, and could have potentially missed some species-specific genes. Furthermore, this study has certain limitations: the differential expression analysis was based on transcriptome data and requires qRT-PCR validation; the functions of candidate genes (e.g., *RpedGSTt-1c* and *RpedCYP4GY1b*) need to be verified through RNA interference or heterologous expression experiments; and the epigenetic mechanisms underlying the intergenerational effects also require further investigation. Recent studies have shown that sublethal insecticide exposure can induce epigenetic imprints in insects, including DNA methylation, histone modification, and non-coding RNA expression changes, and these imprints can be heritable [36]. In the future, techniques such as whole-genome bisulfite sequencing or ChIP-seq could be combined to analyze the regulatory mechanisms of intergenerational expression of detoxification enzyme genes in *R. pedestris* [17]. Nevertheless, this study provides important transcriptomic resources for understanding the metabolic adaptation of *R. pedestris* to thiamethoxam. Although the transcriptomes of several soybean pests have already been sequenced [41,42,43], our findings emphasize the need for continued attention to species-specific molecular responses under insecticide stress. Moreover, while our data clearly demonstrated intergenerational transcriptional reprogramming, the proposed underlying mechanisms (e.g., DNA methylation, histone modification, or small RNAs) are based on evidence from other insect systems and will need to be directly investigated in *R. pedestris* in future studies.

This study provides important transcriptomic resources for understanding the metabolic adaptation of *R. pedestris* to thiamethoxam. For pest management, the key detoxification genes identified here (*RpedGSTt-1c* and *RpedCYP4GY1b*) and their intergenerational induction patterns highlight the risk of accelerated resistance development under sublethal exposure scenarios. These findings support the implementation of the following resistance management tactics in soybean fields: (1) avoiding sublethal doses through precise application technologies; (2) incorporating these candidate genes into molecular resistance monitoring protocols to generate early warnings; and (3) exploring RNAi-based suppression of detoxification genes as a potential adjunct to chemical control. Such integrative approaches, grounded in a mechanistic understanding of metabolic adaptation, are essential for sustaining the efficacy of neonicotinoid insecticides against *R. pedestris* and other soybean pests in the face of escalating selection pressure [44,45].

## 5. Conclusions

This study identified 48 CCEs, 82 CYPs, and 18 GSTs in *R. pedestris*, and revealed significant intergenerational differences in detoxification gene expression patterns under sublethal thiamethoxam stress. In the parental generation, *RpedGSTt-1c* (Theta family GST) was consistently upregulated at all concentrations, *RpedCYP6LV19f* (CYP3 family) was upregulated at low and high concentrations, and *RpedCYP4HA1c* (CYP4 family) was downregulated; in the filial generation, detoxification genes were predominantly downregulated, with *RpedCYP4GY1d/b* (CYP4 family) being the only co-upregulated gene; the genes upregulated in the parental generation were not also upregulated in the filial generation. Direct comparisons showed that the medium concentration (LC_30_) induced the highest number of intergenerationally upregulated genes, exhibiting a non-linear response pattern. These results indicate that parental thiamethoxam exposure leaves an intergenerational transcriptional imprint in the offspring, and the transmission pattern is not a simple replication of the parental response and involves transcriptional reprogramming. This study provides transcriptomic evidence for understanding metabolic adaptation and identified candidate gene expression patterns that may underlie intergenerational tolerance, though direct evidence for thiamethoxam resistance evolution requires further phenotypic validation in *R. pedestris*, which could have important reference value for field resistance monitoring and rational insecticide application.

## Figures and Tables

**Table 1:** Summary of CCE, CYP, and GST gene families identified in *R. pedestris*.

Gene Family	Number Identified	Number with Complete ORF	Homology Range (%)
CCEs	48	21	25.00–94.81
CYPs	82	42	26.61–100.00
GSTs	18	3	44.63–100.00

**Table 2:** Main up- and downregulated detoxification genes in F_0_ and F_1_ generations of *R. pedestris*.

Gene	Family	Putative Function	Generation	Treatment (vs. Control)	Regulation
*RpedGSTt-1c*	GST (Theta)	Xenobiotic detoxification	F_0_	LC_10_, LC_30_, LC_50_	Up
*RpedCYP6LV19f*	CYP (CYP3)	Insecticide metabolism	F_0_	LC_10_, LC_50_	Up
*RpedCYP4HA1c*	CYP (CYP4)	Endogenous metabolism/stress	F_0_	LC_30_, LC_50_	Down
*RpedCYP4GY1d*	CYP (CYP4)	Oxidative stress response	F_1_	LC_10_ (C1 vs. A1)	Up
*RpedCYP4GY1b*	CYP (CYP4)	Oxidative stress response	F_1_	LC_30_, LC_50_ (C2, C3 vs. A1)	Up
*RpedGSTs-1b*	GST (Sigma)	Antioxidant defense	F_1_	All concentrations (C1–C3 vs. A1)	Down
*RpedCYP3225B3i*	CYP (CYP3)	Insecticide metabolism	F_1_	All concentrations (C1–C3 vs. A1)	Down

**Table 3:** Summary of top enriched GO terms and KEGG pathways for DEGs.

Enrichment	Comparison	Description	Rich Factor	Q Value
GO Enrichment	B1 vs. A1	Cytosolic ribosome	3.651535581	0.00011364
	B2 vs. A1	Inner mitochondrial membrane protein complex	3.217357369	0.000112456
	B3 vs. A1	Negative regulation of potassium ion transport	7.665591194	0.000111971
	C1 vs. A1	Myofibril	1.916168498	0.000128835
	C2 vs. A1	Ribosome assembly	3.229383287	0.000152584
	C3 vs. A1	Sperm fibrous sheath	24.21241275	0.0003272
	C1 vs. B1	Response to organic cyclic compound	1.806226787	0.000186915
	C2 vs. B2	Kininogen binding	9.425492611	0.00020832
	C3 vs. B3	Protein-phosphocysteine-sugar phosphotransferase activity	30.76990083	0.000187488
KEGG Enrichment	B1 vs. A1	Synthesis and degradation of ketone bodies	8.01969697	0.779043471604732
	B2 vs. A1	Oxidative phosphorylation	2.220556827	0.0267264741951702
	B3 vs. A1	Toll and Imd signaling pathway	7.959398496	0.0065629789798165
	C1 vs. A1	Cardiac muscle contraction	2.949687683	0.000567803532784179
	C2 vs. A1	Cardiac muscle contraction	3.207541663	0.000182787
	C3 vs. A1	Cardiac muscle contraction	5.363068515	0.00181944534685096
	C1 vs. B1	ECM–receptor interaction	3.214842904	0.00113363408307969
	C2 vs. B2	Cardiac muscle contraction	3.128708421	0.00101755201147293
	C3 vs. B3	Carbon metabolism	1.801001232	0.000223995734741934

## Data Availability

The data are contained within the article. Raw RNA-seq data are available in NCBI SRA (BioProject: PRJNA785271). Processed data are in the Supplementary Materials.

## Supplementary Materials

The following supporting information can be downloaded at: https://www.mdpi.com/article/10.3390/insects17060648/s1, Figure S1–S3: Scatter plot of GO enrichment analysis for differentially expressed genes in B vs. A; Figure S4–S6: Scatter plot of GO enrichment analysis for differentially expressed genes in C vs. A; Figure S7–S9: Scatter plot of GO enrichment analysis for differentially expressed genes in C vs. B; Figure S10–S12: Scatter plot of KEGG enrichment analysis for differentially expressed genes in B vs. A; Figure S13–S15: Scatter plot of KEGG enrichment analysis for differentially expressed genes in C vs. A; Figure S16–S18: Scatter plot of KEGG enrichment analysis for differentially expressed genes in C vs. B. Table S1: Summary of RNA seq data quality per library; Table S2: Summary of transcriptome assembly and functional annotation; Table S3: Assessment of sequencing data quality for downstream differential expression analysis; Table S4: Summary of *R. pedestris* CCEs, CYPs, and GSTs including enzyme families, gene IDs, E-values, ORFs, full lengths, and NCBI Blast results.
