# Supplementary material for: Comparative Transcriptomic Analysis of Detoxification Enzyme Gene Families in Parent and Offspring Riptortus pedestris After Sublethal Thiamethoxam Treatment
Source: Insects. 2026 Jun 19;17(6):648. doi: 10.3390/insects17060648 (PMC13301825; doi:10.3390/insects17060648)
Supplement: Supplementary file 1 [file insects-17-00648-s001.zip › insects-4345997-supplementary.pdf]

Supplementary Materials

Table S1. Summary of RNA-seq data quality per library

Library	Raw reads (M)	Clean reads (M)	Clean reads (%)	Q20 (%)	Q30 (%)	GC (%)	Mapping rate (%)
A1_1	53.13	52.22	98.3	98.9	95.6	45.7	91.51
A1_2	57.88	56.99	98.5	98.9	95.8	46	91.06
A1_3	55.08	54.12	98.3	98.8	95.4	43	90.64
B1_1	60.93	60.01	98.5	98.9	95.8	44.9	90.89
B1_2	43.04	42.07	97.8	98.5	94.7	43.9	91.64
B1_3	46.32	44.94	97	98.5	94.9	47.2	90.86
B2_1	53.53	52.23	97.6	98.5	94.8	43.6	90.87
B2_2	47.24	46.1	97.6	98.5	94.9	43.2	92.05
B2_3	52.57	51.42	97.8	98.5	94.7	48.4	96.15
B3_1	49.97	48.78	97.6	98.5	94.8	46.6	95.93
B3_2	49.97	48.85	97.8	98.4	94.6	43.7	91.31
B3_3	45.07	44.01	97.7	98.4	94.3	41.2	91.5
C1_1	39.71	38.25	96.3	98.3	93.9	41.1	90.39
C1_2	40.86	39.24	96	98.3	94.1	42.6	90.98
C1_3	39.19	37.46	95.6	98.3	94.1	43.3	91.06
C2_1	40.62	39.04	96.1	98.4	94.3	42.8	91.52
C2_2	229.65	220.88	96.2	98.3	94.1	43.3	91.75
C2_3	42.4	40.62	95.8	98.4	94.1	38.6	90.2
C3_1	41.03	39.4	96	98.2	93.7	40.2	90.65
C3_2	40.12	38.52	96	98.2	93.7	39.2	91.43
C3_3	38.87	37.31	96	98.2	93.8	42.4	92.13
Mean	52.9	51.84	97.1	98.5	94.7	43.2	91.98
Range	38.9–229.7	37.3–220.9	95.6–98.5	98.2–98.9	93.7–95.8	38.6–48.4	90.2–96.2

Table S2. Summary of transcriptome assembly and functional annotation

Category	Item	Value
Transcript assembly	Total number of transcripts	679,731
	Total number of unigenes	385,902
	Unigene N50 (bp)	504
	Unigene N90 (bp)	227
	Maximum unigene length (bp)	30,273
	Minimum unigene length (bp)	201
	Unigenes ≥ 500 bp	78,893 (20.44%)
	Unigenes ≥ 1,000 bp	26,790 (6.94%)
	Total unigene length (bp)	174,604,352
	Average unigene length (bp)	452.46
Functional annotation	Annotated in NR	94,143 (24.40%)
	Annotated in NT	80,985 (20.99%)
	Annotated in GO	54,942 (14.24%)
	Annotated in KOG	40,138 (10.40%)
	Annotated in KEGG	32,970 (8.54%)
	Annotated in PFAM	34,574 (8.96%)
	Annotated in CDD	30,580 (7.92%)
	Annotated in at least one database	139,173 (36.06%)
	Annotated in all databases	0 (0%)

Table S3. Assessment of sequencing data quality for downstream differential expression analysis

Quality Metric	Value Obtained	Acceptable Threshold	Meets Standard
Clean Q30 (%)	94.70 ± 0.78	> 85	Yes
Clean Q20 (%)	98.49 ± 0.22	> 90	Yes
GC content (%)	43.19 ± 2.37	35–55	Yes
Clean reads retention rate (%)	97.05 ± 0.94	> 85	Yes
Unigene N50 (bp)	504	> 300	Yes
Unigene N90 (bp)	227	> 200	Yes
Average unigene length (bp)	452.46	> 300	Yes
Total mapping rate (%)	91.51 ± 1.44	> 70	Yes
Unique mapping rate (%)	27.84 ± 13.12	Acceptable	Yes
Annotation rate (≥1 database, %)	36.06	> 30	Yes
NR annotation rate (%)	24.4	> 20	Yes
Overall conclusion	All quality metrics meet the standards for reliable downstream differential expression analysis.		

Table S4. Summary of *R. pedestris* CCEs, CYPs, and GSTs including enzyme families, gene IDs, E-values, ORFs, full lengths, and NCBI Blast results.

Gene Name	Complete ORF	ORF (aa)	BLASTxannotation	Query Cover	E value	Per. Ident	Family
RpedB-Esterase-2a	YES	269	venom carboxylesterase-6 [*Halyomorpha halys*]	99%	8e-67	39.05%	Cces
RpedB-Esterase-16	YES	500	juvenile hormone esterase-like [*Halyomorpha halys*]	99%	0	51.45%	Cces
RpedB-Esterase-37b	Partial	533	juvenile hormone esterase-like [*Halyomorpha halys*]	98%	3e-167	48.13%	Cces
RpedB-Esterase-20b	Partial	496	juvenile hormone esterase-like [*Halyomorpha halys*]	98%	5e-160	48.30%	Cces
RpedB-Esterase-22a	YES	535	esterase 1 [*Arma chinensis*]	98%	8e-165	45.14%	Cces
RpedGlutactin-2a	Partial	397	juvenile hormone esterase [*Halyomorpha halys*]	98%	4e-159	53.11%	Cces
RpedAcetylcholinesterase-1c	YES	542	juvenile hormone esterase [*Halyomorpha halys*]	100%	0	51.27%	Cces
RpedB-Esterase-17b	Partial	366	esterase 1 [*Arma chinensis*]	100%	9e-120	49.46%	Cces
RpedB-Esterase-27	YES	454	esterase 1 [*Arma chinensis*]	98%	8e-174	54.48%	Cces
RpedB-Esterase-42c	Partial	364	juvenile hormone esterase [*Halyomorpha halys*]	99%	4e-167	53.94%	Cces
RpedB-Esterase-26	YES	565	esterase FE4 [*Halyomorpha halys*]	98%	0	56.19%	Cces
RpedGlutactin-1	YES	470	juvenile hormone esterase [*Halyomorpha halys*]	98%	1e-171	51.09%	Cces
RpedB-Esterase-14a	Partial	246	juvenile hormone esterase-like [*Halyomorpha halys*]	98%	2e-95	56.43%	Cces
RpedB-Esterase-2b	Partial	382	juvenile hormone esterase [*Halyomorpha halys*]	100%	2e-124	48.21%	Cces
RpedB-Esterase-37a	Partial	562	venom carboxylesterase-6-like [*Rhodnius prolixus*]	98%	4e-178	46.35%	Cces
RpedB-Esterase-3c	YES	443	carboxylesterase E10 [*Subpsaltria yangi*]	98%	1e-125	39.24%	Cces
RpedB-Esterase-21	YES	557	esterase FE4 [*Halyomorpha halys*]	98%	0	51.45%	Cces
RpedB-Esterase-25a	YES	592	juvenile hormone esterase-like isoform X1 [*Rhodnius prolixus*]	99%	0	56.80%	Cces
RpedB-Esterase-29	YES	532	carboxylesterase [*Eocanthecona furcellata*]	100%	0	56.82%	Cces
RpedB-Esterase-9a	YES	559	esterase E4-like [*Homalodisca vitripennis*]	98%	9e-130	36.83%	Cces
RpedB-Esterase-42a	Partial	201	juvenile hormone esterase [*Halyomorpha halys*]	100%	2e-68	54.41%	Cces
RpedB-Esterase-3a	Partial	169	venom carboxylesterase-6-like [*Myzus persicae*]	99%	7e-59	54.29%	Cces
RpedB-Esterase-20a	Partial	251	esterase E4-like isoform X1 [*Sipha flava*]	99%	2e-68	47.08%	Cces
RpedB-Esterase-20c	YES	587	acetylcholinesterase [*Halyomorpha halys*]	98%	0	83.39%	Cces
RpedB-Esterase-33	YES	482	juvenile hormone esterase, partial [*Bemisia tabaci*]	100%	4e-117	40.12%	Cces
RpedB-Esterase-1b	Partial	582	neuroligin-4, X-linked [*Halyomorpha halys*]	99%	0	87.59%	Cces
RpedB-Esterase-7	Partial	439	neuroligin-4, X-linked [*Halyomorpha halys*]	99%	0	88.10%	Cces
RpedB-Esterase-31	Partial	606	liver carboxylesterase 1 [*Rhodnius prolixus*]	98%	0	57.24%	Cces
RpedB-Esterase-11	Partial	981	neuroligin-1-like isoform X2 [*Cimex lectularius*]	99%	0	47.21%	Cces

RpedB-Esterase-9c	Partial	344	juvenile hormone esterase [*Halyomorpha halys*]	100%	4e-132	54.96%	Cces
RpedB-Esterase-3d	Partial	490	neuroligin-4, Y-linked [*Halyomorpha halys*]	100%	0	91.46%	Cces
RpedB-Esterase-22b	Partial	322	juvenile hormone esterase [*Halyomorpha halys*]	98%	4e-128	58.51%	Cces
RpedB-Esterase-39a	YES	766	neurotactin [*Cimex lectularius*]	98%	0	47.86%	Cces
RpedB-Esterase-25b	Partial	359	neuroligin-4, X-linked-like isoform X4 [*Rhodnius prolixus*]	99%	0	72.49%	Cces
RpedAcetylcholinesterase-1a	Partial	325	acetylcholinesterase-like isoform X1 [*Rhodnius prolixus*]	100%	0	80.37%	Cces
RpedB-Esterase-42b	Partial	197	neuroligin-4, Y-linked-like [*Cimex lectularius*]	98%	1e-117	90.21%	Cces
RpedB-Esterase-45b	YES	401	acetylcholinesterase [*Halyomorpha halys*]	100%	0	70.02%	Cces
RpedB-Esterase-1a	YES	306	juvenile hormone esterase-like [*Rhodnius prolixus*]	98%	7e-59	36.62%	Cces
RpedB-Esterase-15	YES	100	esterase E4-like isoform X2 [*Lycorma delicatula*]	98%	2e-09	25.00%	Cces
RpedB-Esterase-2d	Partial	170	juvenile hormone esterase-like [*Halyomorpha halys*]	99%	5e-48	42.25%	Cces
RpedB-Esterase-9b	Partial	61	juvenile hormone esterase-like [*Halyomorpha halys*]	98%	1e-25	40.37%	Cces
RpedB-Esterase-13b	YES	403	carboxylesterase 4A [*Rhodnius prolixus*]	98%	1e-123	48.66%	Cces
RpedB-Esterase-38	Partial	172	juvenile hormone esterase [*Halyomorpha halys*]	99%	7e-37	39.27%	Cces
RpedB-Esterase-13a	Partial	156	juvenile hormone esterase-like [*Halyomorpha halys*]	99%	1e-60	57.14%	Cces
RpedB-Esterase-3b	Partial	204	juvenile hormone esterase [*Halyomorpha halys*]	98%	9e-50	42.74%	Cces
RpedAcetylcholinesterase-1b	Partial	341	neuroligin-4, Y-linked [*Halyomorpha halys*]	100%	0	94.81%	Cces
RpedB-Esterase-14b	YES	372	neuroligin-2 [*Halyomorpha halys*]	99%	0	82.97%	Cces
RpedAcetylcholinesterase-1e	YES	82	neuroligin-2 [*Halyomorpha halys*]	98%	2e-41	87.85%	Cces
RpedCYP6-1b	Partial	262	cytochrome P450 6k1 [*Bemisia tabaci*]	98%	2e-67	42.32%	Cyps
RpedCYP6-1a	Partial	194	cytochrome P450 [*Nesidiocoris tenuis*]	98%	2e-69	56.48%	Cyps
RpedCYP6LU1	YES	506	cytochrome P450 [*Nesidiocoris tenuis*]	98%	1e-147	46.17%	Cyps
RpedCYP3092E3a	YES	517	cytochromeP450 [*Riptortus pedestris*]	99%	2e-176	46.89%	Cyps
RpedCYP395R2b	YES	515	cytochromeP450 [*Riptortus pedestris*]	99%	0	71.12%	Cyps
RpedCYP6LV19a	YES	428	cytochrome P450 6a2-like [*Halyomorpha halys*]	98%	8e-147	49.89%	Cyps
RpedCYP395R2a	YES	510	cytochromeP450 [*Riptortus pedestris*]	100%	0	99.80%	Cyps
RpedCYP395R1a	YES	510	cytochromeP450 [*Riptortus pedestris*]	99%	1e-145	42.32%	Cyps
RpedCYP395R1b	Partial	513	cytochromeP450 [*Riptortus pedestris*]	100%	6e-148	43.39%	Cyps
RpedCYP395P1b	YES	517	cytochromeP450 [*Riptortus pedestris*]	98%	2e-146	42.11%	Cyps
RpedCYP6LT2c	YES	329	cytochrome P450 6a13 [*Arma chinensis*]	98%	1e-116	48.52%	Cyps
RpedCYP395P1a	YES	515	cytochromeP450 [*Riptortus pedestris*]	99%	6e-161	43.77%	Cyps
RpedCYP395P1c	YES	480	cytochromeP450 [*Riptortus pedestris*]	99%	7e-160	46.33%	Cyps
RpedCYP3231A1	YES	466	cytochromeP450 [*Riptortus pedestris*]	100%	0	99.79%	Cyps

RpedCYP3227A1	YES	501	cytochrome P450 6a2 [*Halyomorpha halys*]	99%	2e-168	48.50%	Cyps
RpedCYP6LT2b	Partial	193	cytochrome P450 6a14 [*Arma chinensis*]	99%	8e-123	53.29%	Cyps
RpedCYP3090C1b	YES	500	cytochrome P450 family 6, partial [*Riptortus pedestris*]	100%	0	100.00%	Cyps
RpedCYP3229A1	YES	307	cytochromeP450 [*Riptortus pedestris*]	100%	0%	98.73%	Cyps
RpedCYP3090C1a	Partial	396	cytochrome P450 6j1 [*Halyomorpha halys*]	100%	0	63.73%	Cyps
RpedCYP395P1d	Partial	357	cytochrome P450 6a17 [*Geocoris pallidipennis*]	99%	2e-162	56.06%	Cyps
RpedCYP395P1e	Partial	435	cytochrome P450 6a17 [*Geocoris pallidipennis*]	100%	1e-159	50.34%	Cyps
RpedCYP6LT2a	Partial	289	cytochrome P450 6a14 [*Arma chinensis*]	100%	9e-101	49.66%	Cyps
RpedCYP3226B1a	YES	375	cytochrome P450 6a14 [*Arma chinensis*]	98%	4e-60	31.68%	Cyps
RpedCYP3226B1b	Partial	373	cytochrome P450 6a14 [*Arma chinensis*]	100%	4e-54	30.31%	Cyps
RpedCYP6LV20d	Partial	241	cytochrome P450 6a2 [*Halyomorpha halys*]	100%	8e-96	55.22%	Cyps
RpedCYP395R1c	Partial	235	cytochrome P450 6a13 [*Geocoris pallidipennis*]	99%	2e-102	52.04%	Cyps
RpedCYP3225B3c	YES	378	cytochrome P450 6j1 isoform X1 [*Halyomorpha halys*]	98%	4e-78	35.66%	Cyps
RpedCYP6-1c	YES	177	cytochrome P450 6k1-like isoform X2 [*Melanaphis sacchari*]	98%	4e-54	51.14%	Cyps
RpedCYP4HA1d	YES	498	cytochromeP450 [*Riptortus pedestris*]	99%	0	62.88%	Cyps
RpedCYP4HD1b	YES	504	cytochrome P450 4C1 [*Halyomorpha halys*]	99%	0	59.40%	Cyps
RpedCYP4GY1e	YES	488	cytochrome P450 4C1 isoform X1 [*Halyomorpha halys*]	99%	1e-157	47.34%	Cyps
RpedCYP6LV19b	Partial	171	cytochrome P450 monooxygenase CYP6X1v1 [*Lygus lineolaris*]	99%	6e-33	35.48%	Cyps
RpedCYP3225B3d	YES	238	cytochrome P450 6j1 isoform X1 [*Halyomorpha halys*]	99%	4e-57	43.50%	Cyps
RpedCYP4HA1c	YES	492	cytochromeP450 [*Riptortus pedestris*]	100%	0	100.00%	Cyps
RpedCYP4GY1b	YES	486	cytochrome P450 4C1 [*Halyomorpha halys*]	98%	7e-130	41.22%	Cyps
RpedCYP3225B3b	YES	199	cytochrome P450 6j1 [*Halyomorpha halys*]	98%	5e-46	43.38%	Cyps
RpedCYP6LV19e	YES	148	cytochrome P450 6a2-like [*Halyomorpha halys*]	99%	2e-60	58.02%	Cyps
RpedCYP4GY1a	YES	486	cytochrome P450 4C1-like [*Halyomorpha halys*]	98%	2e-137	43.33%	Cyps
RpedCYP4GY1c	YES	484	cytochrome P450 4C1-like [*Halyomorpha halys*]	99%	2e-141	42.83%	Cyps
RpedCYP4GX1	Partial	338	cytochrome P450 4g15-like [*Halyomorpha halys*]	99%	3e-171	68.60%	Cyps
RpedCYP3225B3j	YES	157	cytochrome P450 6k1 [*Halyomorpha halys*]	98%	3e-56	43.98%	Cyps
RpedCYP4G-4a	YES	183	cytochrome P450 4g15-like [*Cimex lectularius*]	99%	4e-122	86.80%	Cyps
RpedCYP3225B3a	Partial	178	cytochrome P450 6k1 [*Halyomorpha halys*]	99%	6e-52	45.74%	Cyps
RpedCYP3225B3i	Partial	256	cytochrome P450 6j1 [*Halyomorpha halys*]	100%	4e-64	38.76%	Cyps
RpedCYP4HA1b	Partial	237	cytochromeP450 [*Riptortus pedestris*]	100%	6e-102	60.92%	Cyps
RpedCYP314A1	YES	510	cytochrome P450 314A1 [*Lygus hesperus*]	98%	0	56.08%	Cyps

RpedCYP6LV19c	Partial	152	cytochrome P450 6a22 [*Halyomorpha halys*]	100%	2e-36	45.81%	Cyps
RpedCYP15A1b	YES	498	cytochrome P450 [*Nesidiocoris tenuis*]	98%	0	77.11%	Cyps
RpedCYP15A1a	YES	498	cytochrome P450 [*Nesidiocoris tenuis*]	98%	0	76.71%	Cyps
RpedCYP3224B2-2a	Partial	491	cytochrome P450 4c21 isoform X2 [*Halyomorpha halys*]	98%	4e-74	33.74%	Cyps
RpedCYP395R1d	Partial	88	cytochrome P450 6a13 [*Geocoris pallidipennis*]	99%	5e-29	43.38%	Cyps
RpedCYP301A1	YES	507	cytochrome P450 [*Nesidiocoris tenuis*]	99%	0	71.18%	Cyps
RpedCYP306A1b	YES	524	cytochrome P450 18a1 [*Rhodnius prolixus*]	99%	0	73.77%	Cyps
RpedCYP307B1	YES	480	cytochrome P450 307a1 [*Halyomorpha halys*]	100%	0	68.12%	Cyps
RpedCYP4GY1d	Partial	417	cytochrome P450 4C1-like [*Halyomorpha halys*]	99%	3e-121	43.90%	Cyps
RpedCYP3223A1c	Partial	486	cytochrome P450 4C1-like isoform X1 [*Rhodnius prolixus*]	99%	1.00E-84	34.13%	Cyps
RpedCYP3224B2-2g	YES	351	cytochrome P450 4c3-like isoform X2 [*Rhodnius prolixus*]	98%	1e-48	31.39%	Cyps
RpedCYP3230A1	Partial	131	cytochrome P450 6k1 [*Halyomorpha halys*]	99%	2e-41	49.35%	Cyps
RpedCYP3224B2-2c	YES	192	cytochrome P450 4c21 [*Halyomorpha halys*]	99%	4e-39	42.00%	Cyps
RpedCYP4HA1a	YES	390	cytochromeP450 [*Riptortus pedestris*]	100%	2e-166	57.80%	Cyps
RpedCYP3224B2-2f	YES	442	cytochrome P450 4C1 isoform X1 [*Halyomorpha halys*]	98%	2e-65	30.80%	Cyps
RpedCYP3224B2-2b	YES	351	cytochrome P450 4C1 isoform X1 [*Halyomorpha halys*]	99%	3e-69	31.39%	Cyps
RpedCYP3223A1d	Partial	271	cytochrome P450 4C1-like isoform X2 [*Rhodnius prolixus*]	99%	6e-57	36.56%	Cyps
RpedCYP3224B2-2d	YES	410	cytochrome P450 4C1 isoform X2 [*Nilaparvata lugens*]	99%	2e-38	26.61%	Cyps
RpedCYP6LV19f	Partial	76	cytochrome P450 6a2 [*Halyomorpha halys*]	99%	3e-18	39.42%	Cyps
RpedCYP3224B2-2m	Partial	145	cytochrome P450 [*Nesidiocoris tenuis*]	99%	1E-35	42.50%	Cyps
RpedCYP4G-3a	Partial	354	cytochrome P450 4g15 [*Halyomorpha halys*]	99%	5e-151	54.23%	Cyps
RpedCYP305L1	YES	492	cytochrome P450 [*Nesidiocoris tenuis*]	99%	0	52.85%	Cyps
RpedCYP3223A1b	Partial	267	cytochrome P450 4c3-like isoform X3 [*Rhodnius prolixus*]	100%	4e-25	29.86%	Cyps
RpedCYP3224B2-2n	Partial	309	cytochrome P450 4c21 [*Halyomorpha halys*]	100%	4e-42	29.46%	Cyps
RpedCYP3223A1a	Partial	369	cytochrome P450 4C1-like isoform X2 [*Rhodnius prolixus*]	99%	2e-39	29.95%	Cyps
RpedCYP3224B2-2h	Partial	372	cytochrome P450 4c21 [*Halyomorpha halys*]	98%	3e-32	26.88%	Cyps
RpedCYP4G-3b	Partial	243	cytochrome P450 4g15 [*Halyomorpha halys*]	100%	3e-113	65.86%	Cyps
RpedCYP3224B2-2e	Partial	333	cytochrome P450 4c21 [*Halyomorpha halys*]	100%	7e-44	28.10%	Cyps
RpedCYP315A1	Partial	258	cytochrome P450 315a1 [*Geocoris pallidipennis*]	100%	3e-146	62.35%	Cyps
RpedCYP3224B2-2L	Partial	247	cytochrome P450 4c3 [*Halyomorpha halys*]	100%	9e-29	31.79%	Cyps
RpedCYP4G-4b	Partial	326	cytochrome P450 4g15 isoform X1 [*Rhodnius prolixus*]	98%	3e-82	42.03%	Cyps
RpedCYP3224B2-2i	Partial	185	cytochrome P450 [*Nesidiocoris tenuis*]	98%	5e-17	28.90%	Cyps
RpedCYP306A1a	Partial	152	cytochrome P450 306a1 isoform X1 [*Halyomorpha halys*]	100%	8e-77	65.84%	Cyps

RpedCYP302A2	Partial	80	cytochrome P450 302a1, mitochondrial [*Rhodnius prolixus*]	98%	3e-54	49.43%	Cyps
RpedCYP3224B2-2k	Partial	177	cytochrome P450 4C1 isoform X2 [*Halyomorpha halys*]	99%	3e-14	26.67%	Cyps
RpedCYP3224B2-2j	Partial	199	cytochrome P450 4c3 [*Halyomorpha halys*]	98%	3e-13	27.57%	Cyps
RpedGSTt-1d	Partial	167	glutathione S-transferase 1-like protein [*Arma chinensis*]	100%	3e-106	77.04%	Gsts
RpedGSTt-1c	YES	187	glutathione S-transferase 1-like [*Rhodnius prolixus*]	98%	1e-72	55.68%	Gsts
RpedGSTz	YES	217	Glutathione S-transferase [*Nesidiocoris tenuis*]	100%	3e-145	88.02%	Gsts
RpedGSTm-2	YES	77	microsomal glutathione S-transferase 1 [*Halyomorpha halys*]	98%	6e-39	72.28%	Gsts
RpedGSTm-1	YES	154	microsomal glutathione s-transferase [*Riptortus pedestris*]	99%	5e-106	100.00%	Gsts
RpedGSTs-5	YES	101	glutathione S-transferase isoform X2 [*Halyomorpha halys*]	99%	9e-57	86.14%	Gsts
RpedGSTm-3	YES	204	glutathione-S-transferase 3, partial [*Geocoris pallidipennis*]	100%	4e-109	74.51%	Gsts
RpedGSTs-1h	YES	203	glutathione S-transferase-like [*Halyomorpha halys*]	100%	3e-81	56.65%	Gsts
RpedGSTs-1e	YES	203	glutathione S-transferase-like [*Halyomorpha halys*]	100%	5e-75	55.17%	Gsts
RpedGSTs-1f	YES	204	glutathione S-transferase-like [*Halyomorpha halys*]	100%	3e-73	56.37%	Gsts
RpedGSTs-7b	YES	209	glutathione S-transferase [*Eocanthecona furcellata*]	100%	2e-81	57.42%	Gsts
RpedGSTs-1a	Partial	171	glutathione S-transferase-like [*Halyomorpha halys*]	99%	3e-53	52.91%	Gsts
RpedGSTs-1g	YES	174	glutathione S-transferase-like [*Halyomorpha halys*]	98%	1e-59	52.11%	Gsts
RpedGSTs-1c	YES	132	glutathione S-transferase-like [*Halyomorpha halys*]	98%	6e-55	52.84%	Gsts
RpedGSTs-7a	Partial	150	glutathione S-transferase [*Eocanthecona furcellata*]	100%	6e-60	61.59%	Gsts
RpedGSTs-1b	YES	98	glutathione S-transferase-like [*Halyomorpha halys*]	98%	5e-27	44.63%	Gsts
RpedGSTt-1b	YES	29	glutathione S-transferase theta-3 [*Halyomorpha halys*]	99%	5e-26	69.12%	Gsts
RpedGSTt-1a	YES	30	glutathione S-transferase theta-1-like [*Cimex lectularius*]	100%	4e-24	52.53%	Gsts

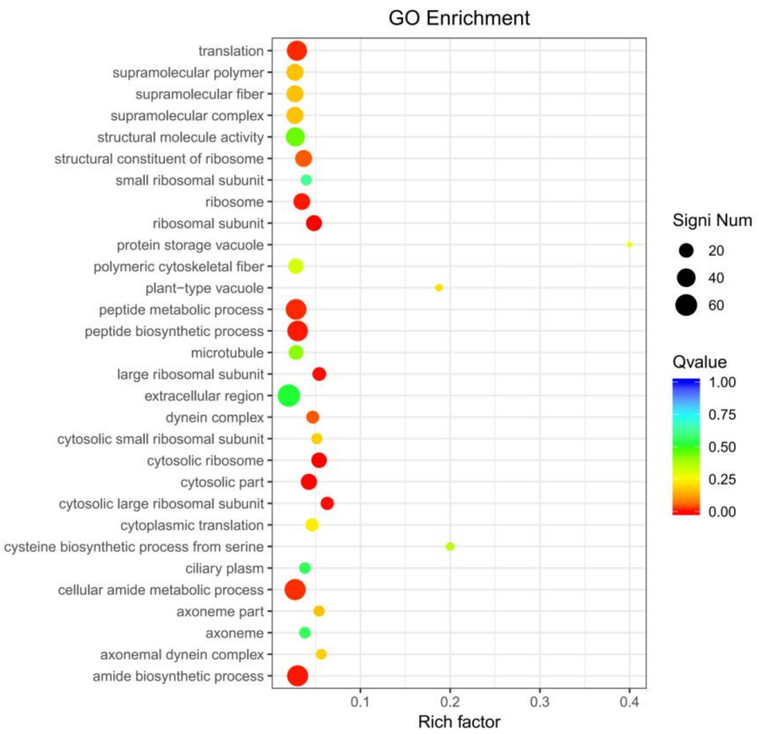

Figure S1 Scatter plot of GO enrichment analysis for differentially expressed genes in B1 vs A1. The x-axis represents the Rich factor, and the y-axis represents GO terms. Dot size indicates the number of differentially expressed genes, and color indicates the significance level of the q-value

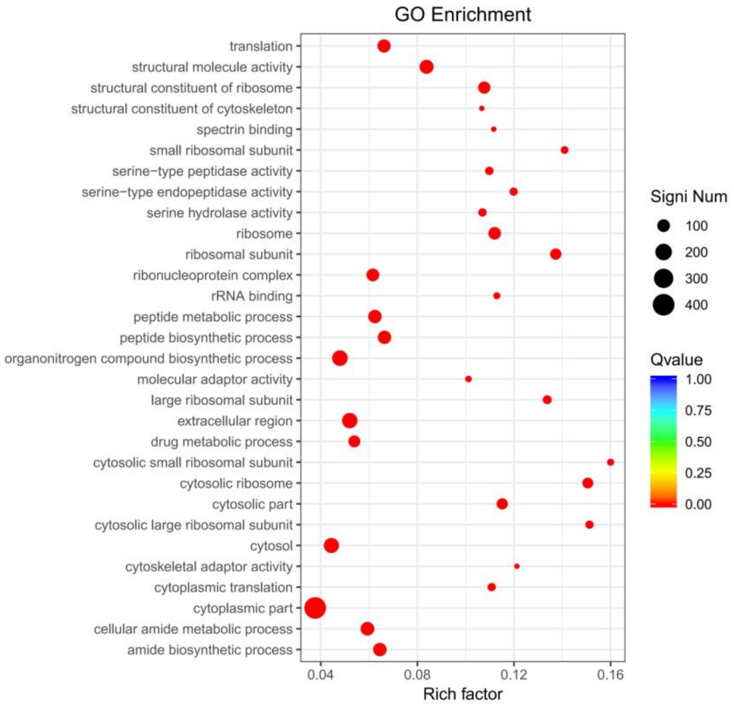

Figure S2 Scatter plot of GO enrichment analysis for differentially expressed genes in B2 vs A1. The x-axis represents the Rich factor, and the y-axis represents GO terms.

Dot size indicates the number of differentially expressed genes, and color indicates the significance level of the q-value.

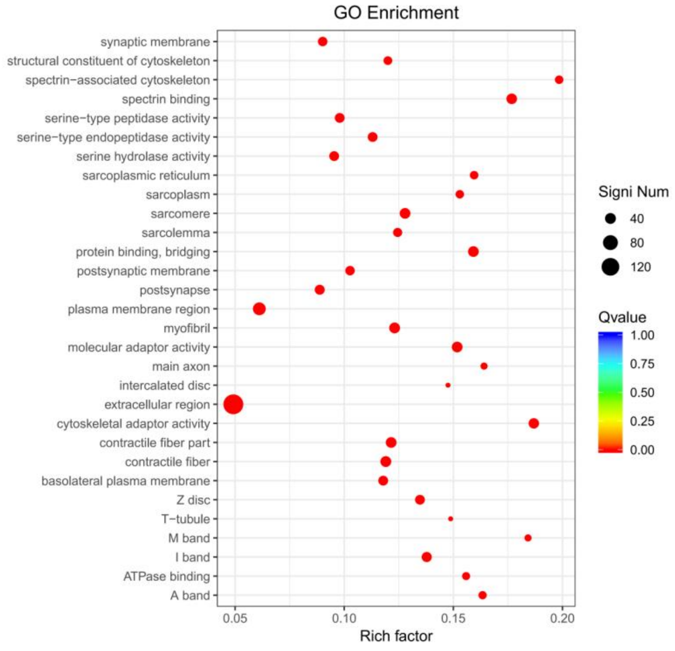

Figure S3 Scatter plot of GO enrichment analysis for differentially expressed genes in B3 vs A1. The x-axis represents the Rich factor, and the y-axis represents GO terms. Dot size indicates the number of differentially expressed genes, and color indicates the significance level of the q-value.

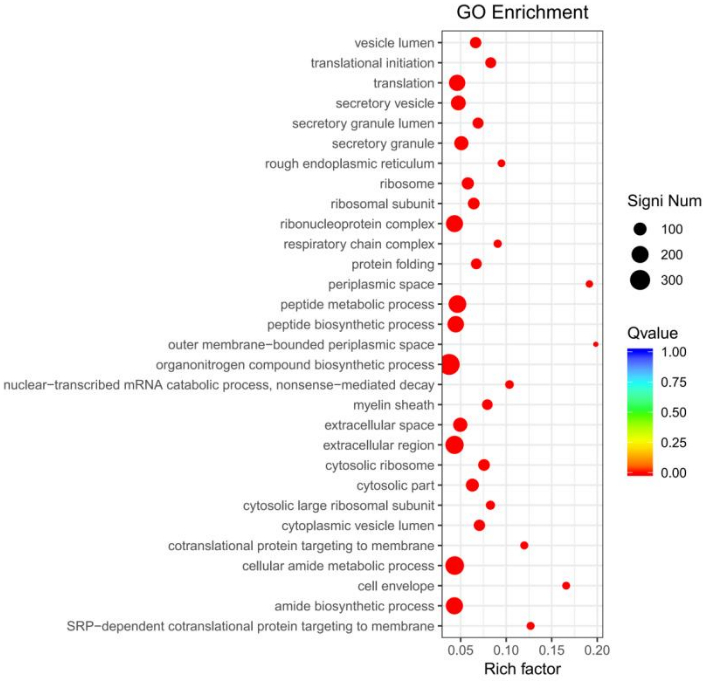

Figure S4 Scatter plot of GO enrichment analysis for differentially expressed genes in C1 vs A1. The x-axis represents the Rich factor, and the y-axis represents GO terms.

Dot size indicates the number of differentially expressed genes, and color indicates the significance level of the q-value.

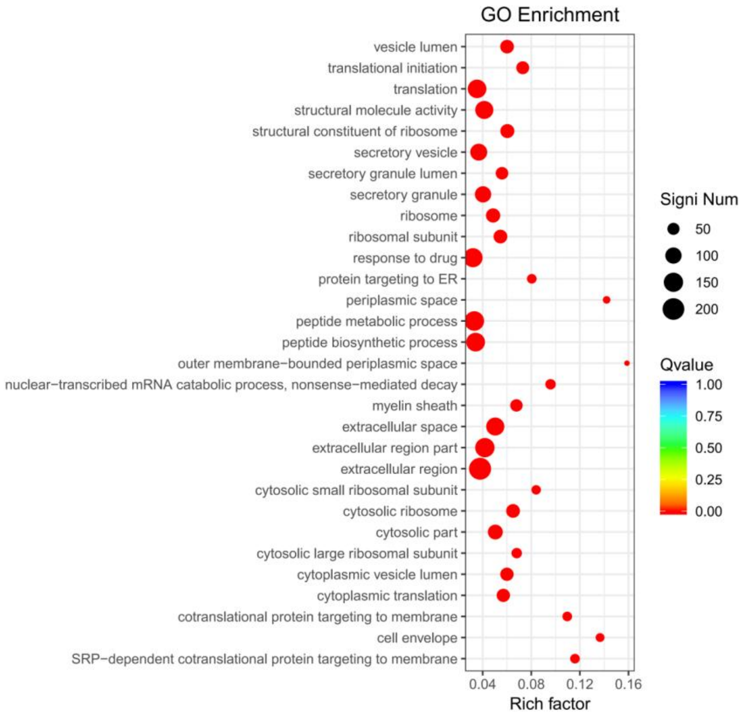

Figure S5 Scatter plot of GO enrichment analysis for differentially expressed genes in C2 vs A1. The x-axis represents the Rich factor, and the y-axis represents GO terms. Dot size indicates the number of differentially expressed genes, and color indicates the significance level of the q-value.

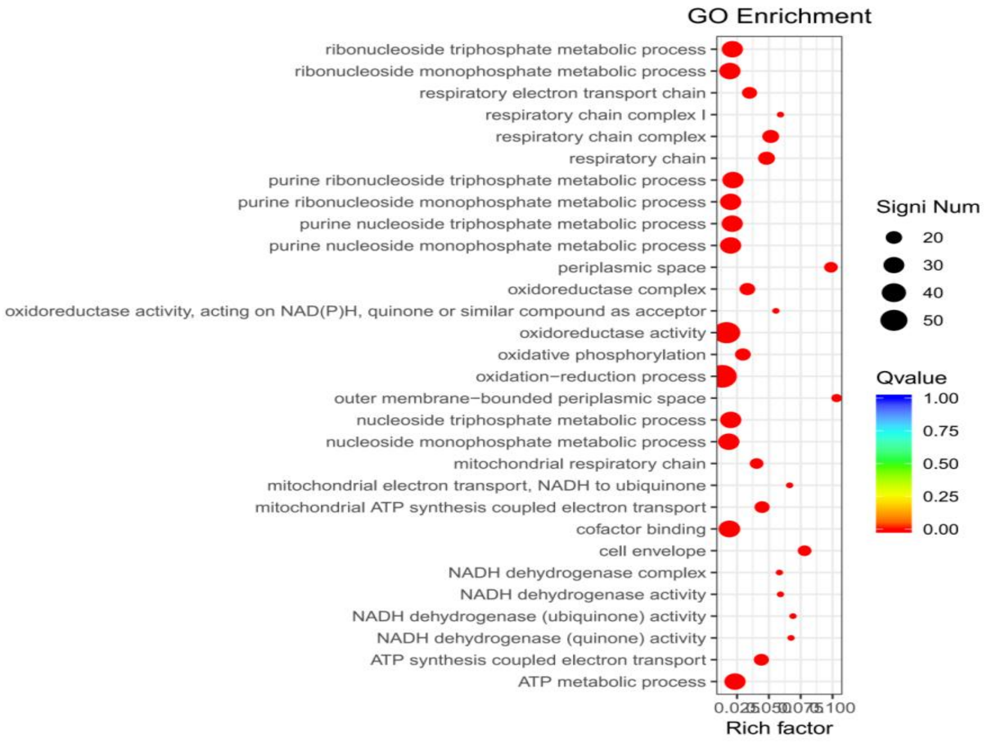

Figure S6 Scatter plot of GO enrichment analysis for differentially expressed genes in C3 vs A1. The x-axis represents the Rich factor, and the y-axis represents GO terms. Dot size indicates the number of differentially expressed genes, and color indicates the significance level of the q-value.

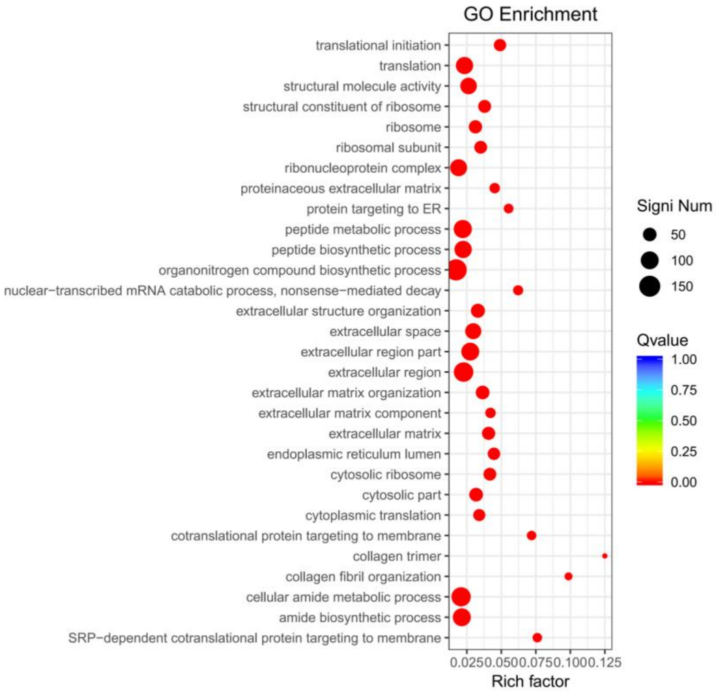

Figure S7 Scatter plot of GO enrichment analysis for differentially expressed genes in C1 vs B1. The x-axis represents the Rich factor, and the y-axis represents GO terms. Dot size indicates the number of differentially expressed genes, and color indicates the significance level of the q-value.

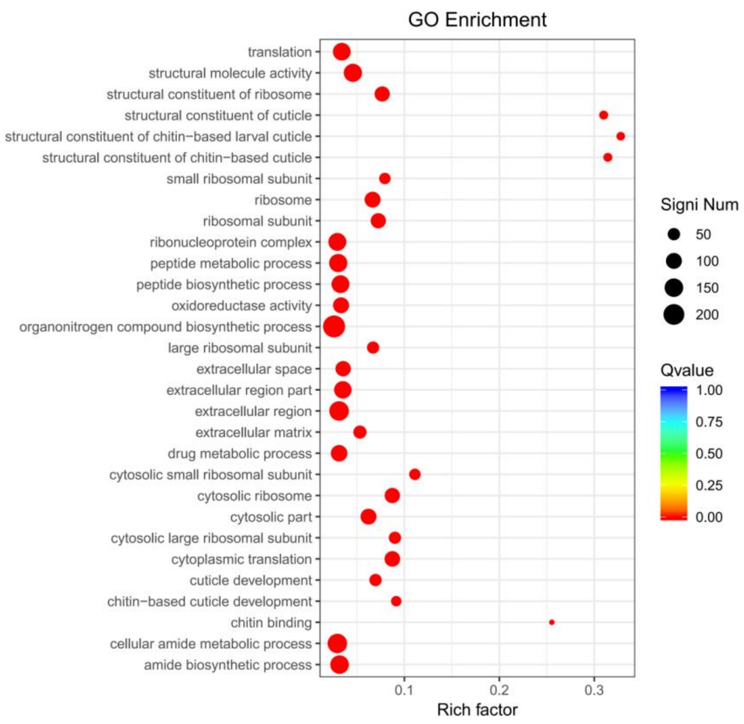

Figure S8 Scatter plot of GO enrichment analysis for differentially expressed genes in C2 vs B2. The x-axis represents the Rich factor, and the y-axis represents GO terms. Dot size indicates the number of differentially expressed genes, and color indicates the significance level of the q-value.

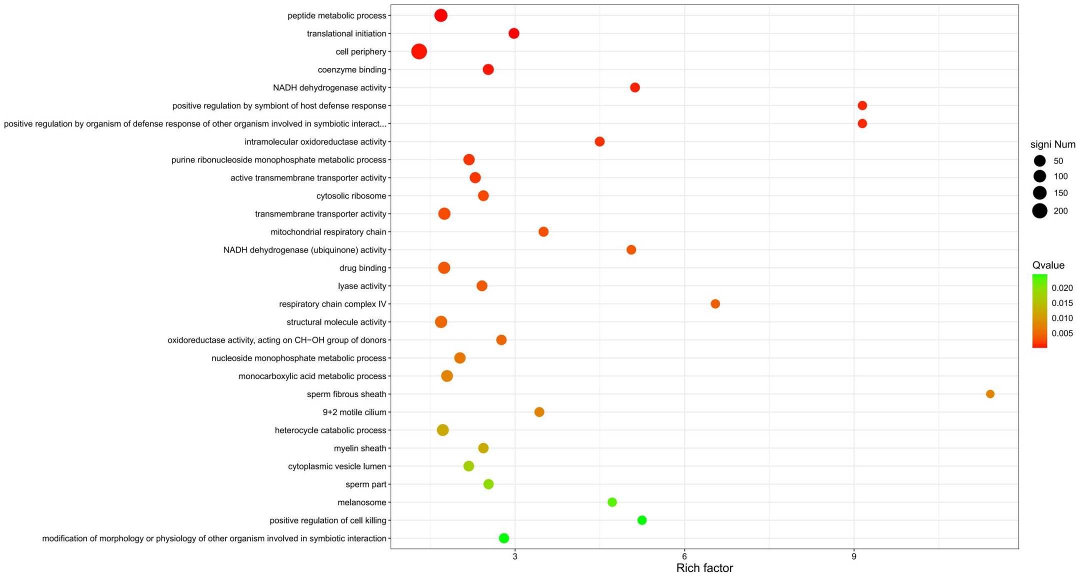

Figure S9 Scatter plot of GO enrichment analysis for differentially expressed genes in C3 vs B3. The x-axis represents the Rich factor, and the y-axis represents GO terms. Dot size indicates the number of differentially expressed genes, and color indicates the significance level of the q-value.

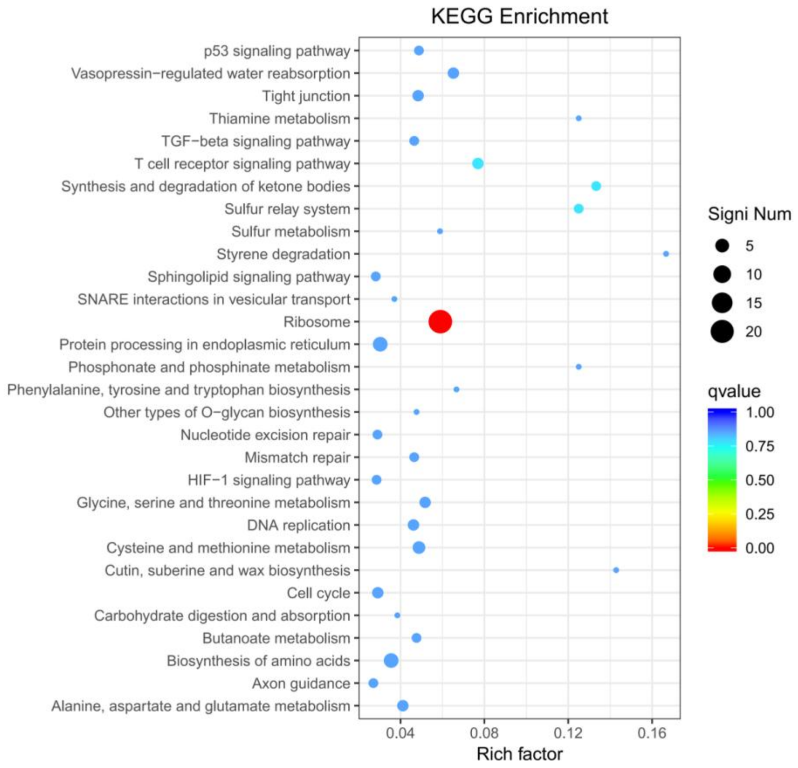

Figure S10 Scatter plot of KEGG enrichment analysis for differentially expressed genes in B1 vs A1. The x-axis represents the Rich factor, and the y-axis represents KEGG pathways. Dot size indicates the number of differentially expressed genes, and color indicates the significance level of the q-value.

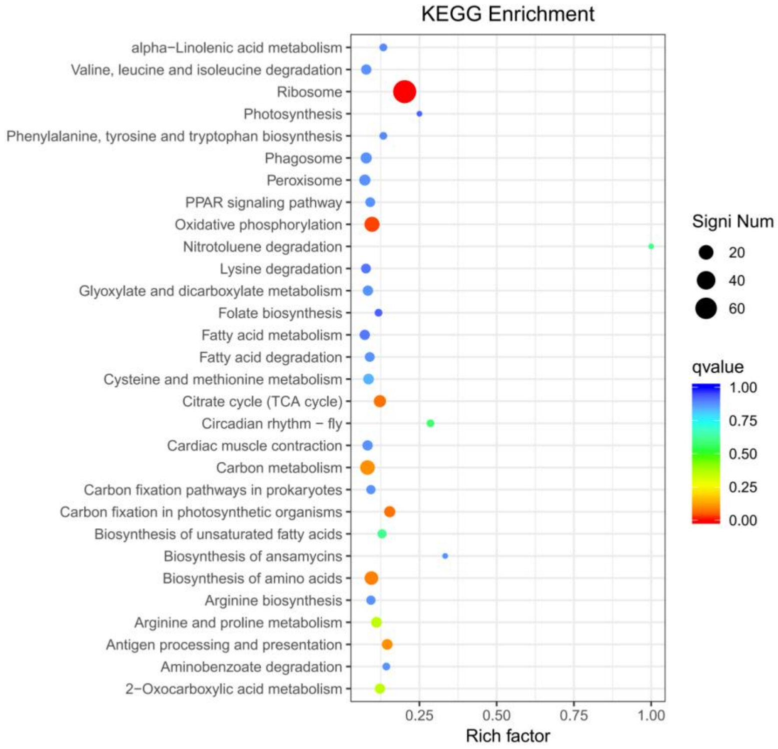

Figure S11 Scatter plot of KEGG enrichment analysis for differentially expressed genes in B2 vs A1. The x-axis represents the Rich factor, and the y-axis represents KEGG pathways. Dot size indicates the number of differentially expressed genes, and color indicates the significance level of the q-value.

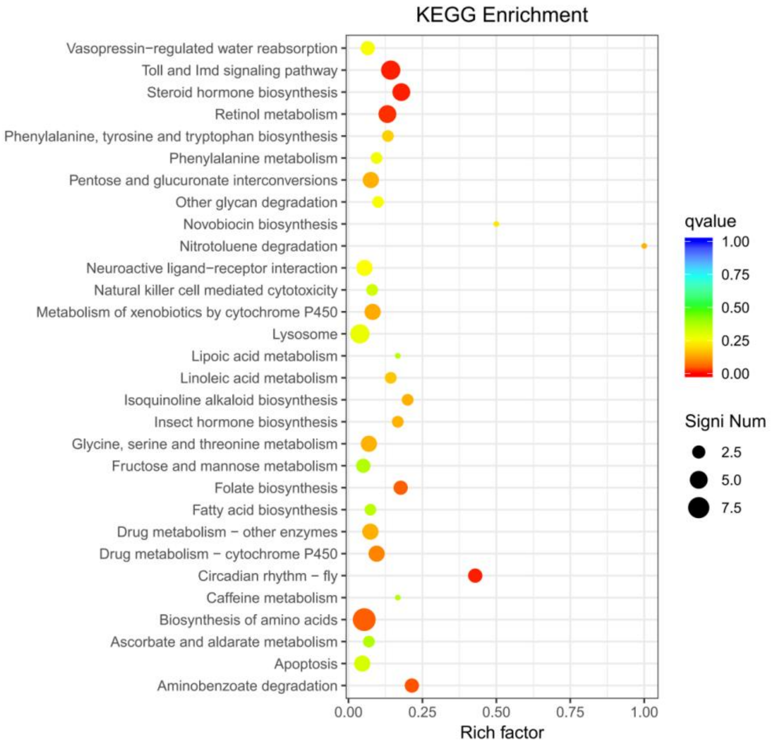

Figure S12 Scatter plot of KEGG enrichment analysis for differentially expressed genes in B3 vs A1. The x-axis represents the Rich factor, and the y-axis represents KEGG pathways. Dot size indicates the number of differentially expressed genes, and color indicates the significance level of the q-value.

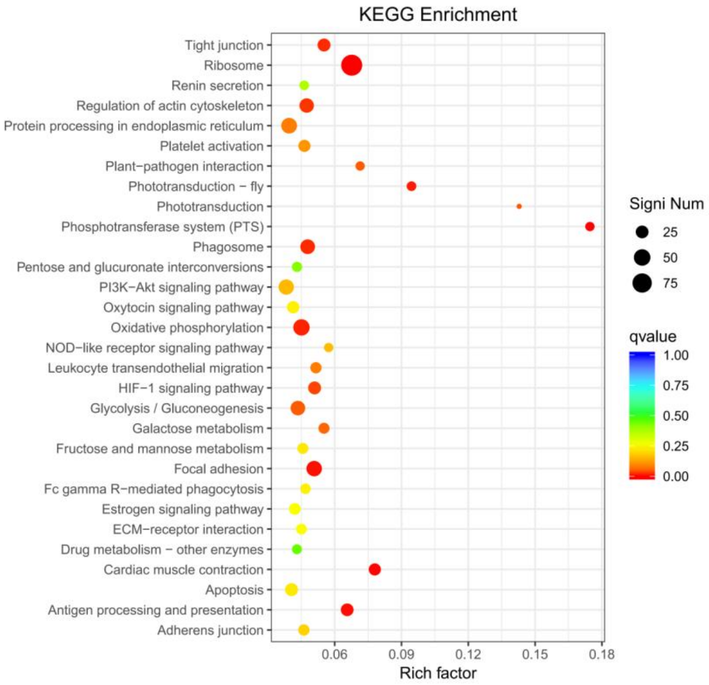

Figure S13 Scatter plot of KEGG enrichment analysis for differentially expressed genes in C1 vs A1. The x-axis represents the Rich factor, and the y-axis represents KEGG pathways. Dot size indicates the number of differentially expressed genes, and color indicates the significance level of the q-value.

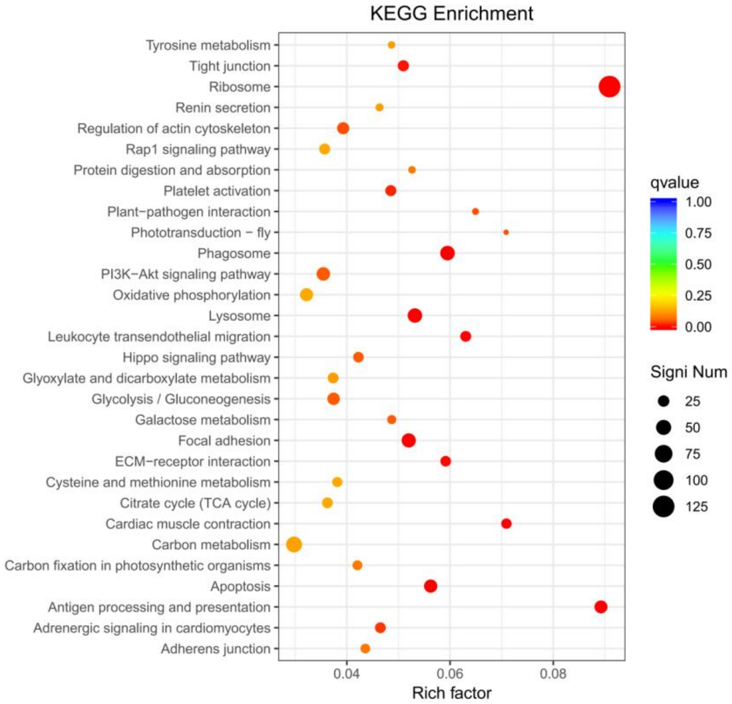

Figure S14 Scatter plot of KEGG enrichment analysis for differentially expressed genes in C2 vs A1. The x-axis represents the Rich factor, and the y-axis represents KEGG pathways. Dot size indicates the number of differentially expressed genes, and

color indicates the significance level of the q-value.

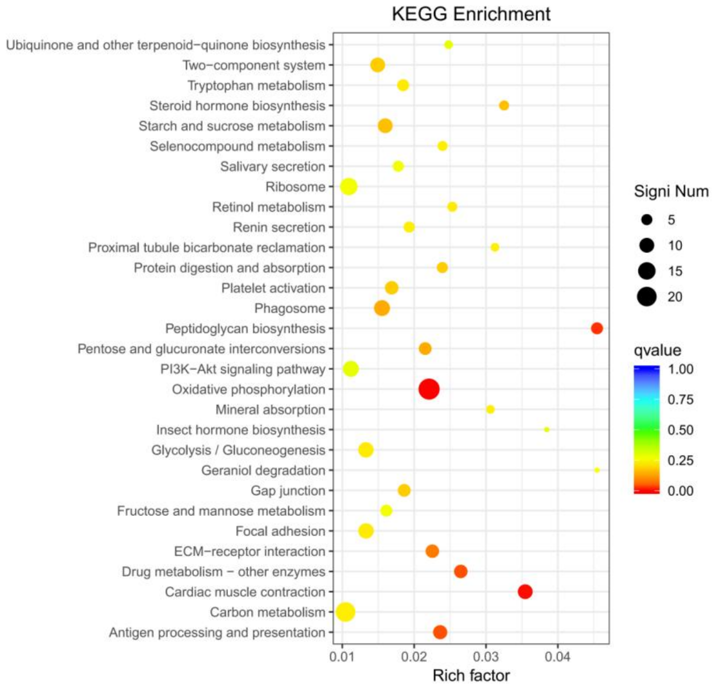

Figure S15 Scatter plot of KEGG enrichment analysis for differentially expressed genes in C3 vs A1. The x-axis represents the Rich factor, and the y-axis represents KEGG pathways. Dot size indicates the number of differentially expressed genes, and color indicates the significance level of the q-value.

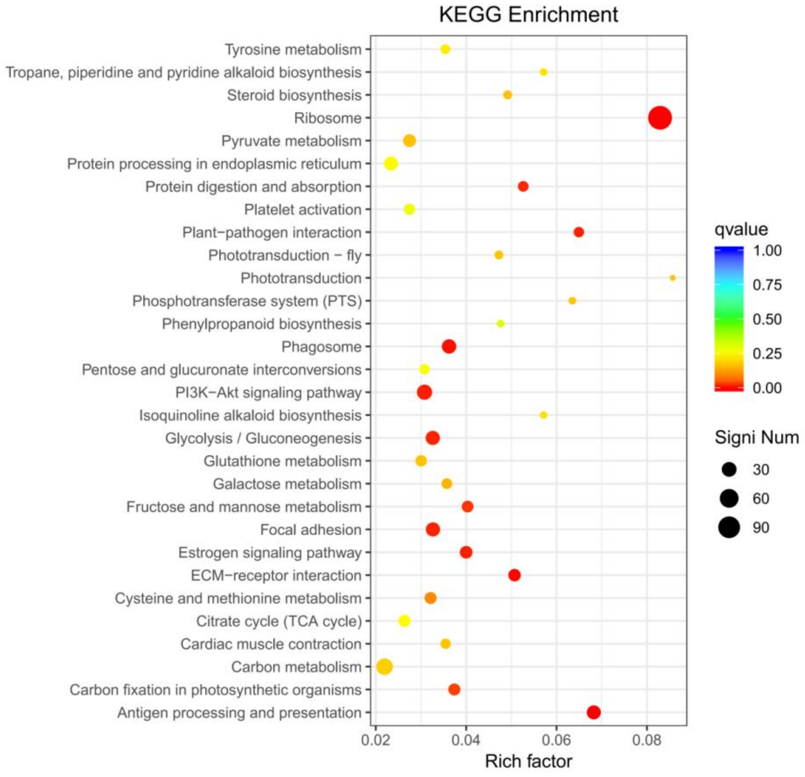

Figure S16 Scatter plot of KEGG enrichment analysis for differentially expressed genes in C1 vs B1. The x-axis represents the Rich factor, and the y-axis represents KEGG pathways. Dot size indicates the number of differentially expressed genes, and color indicates the significance level of the q-value.

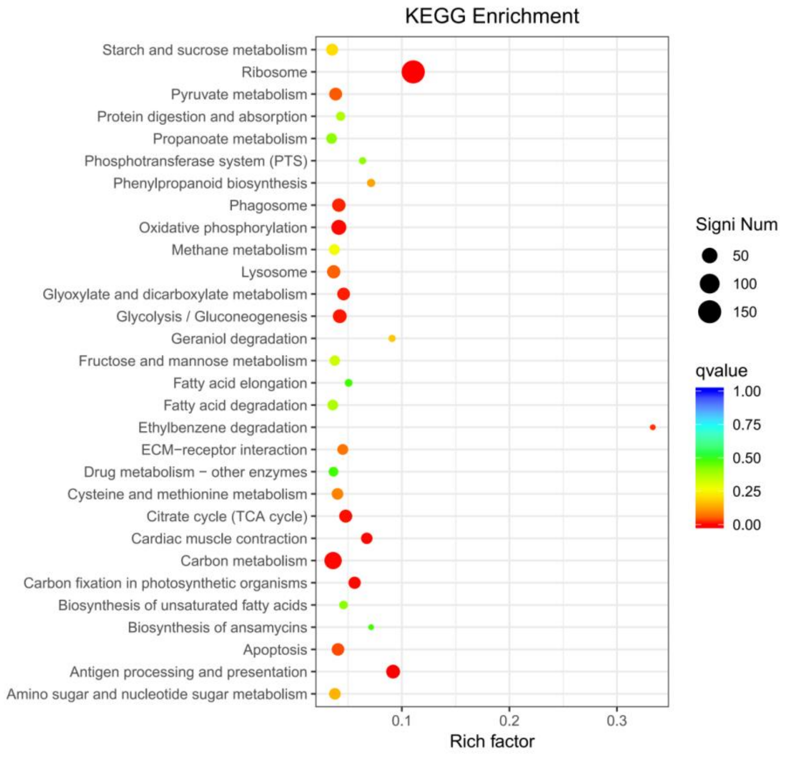

Figure S17 Scatter plot of KEGG enrichment analysis for differentially expressed genes in C2 vs B2. The x-axis represents the Rich factor, and the y-axis represents KEGG pathways. Dot size indicates the number of differentially expressed genes, and color indicates the significance level of the q-value.

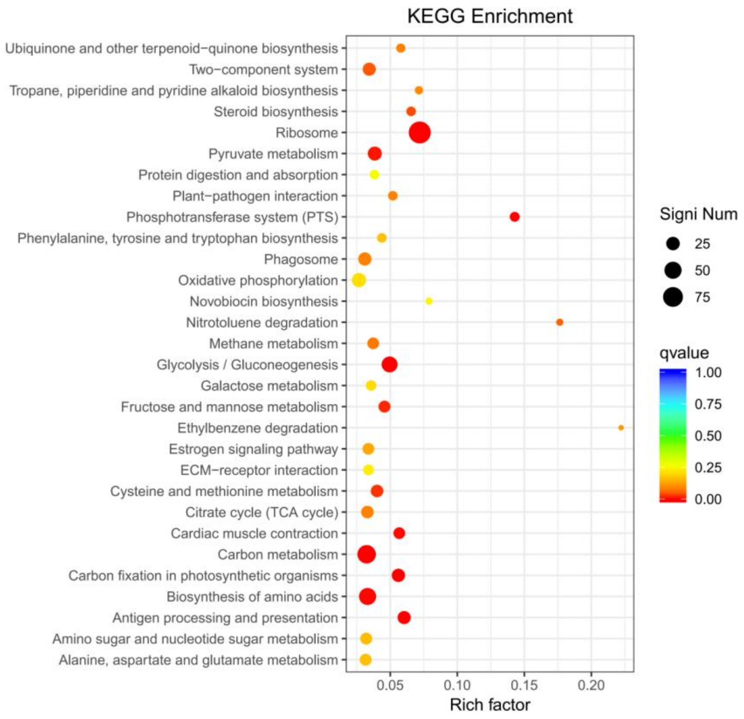

Figure S18 Scatter plot of KEGG enrichment analysis for differentially expressed genes in C3 vs B3. The x-axis represents the Rich factor, and the y-axis represents KEGG pathways. Dot size indicates the number of differentially expressed genes, and color indicates the significance level of the q-value.
